# The gut microbiota–inflammageing axis in cardiometabolic health of older adults and dietary intervention strategies

**DOI:** 10.3389/fnut.2026.1931405

**Published:** 2026-09-22

**Authors:** Xin Liu, Shuo Huang, Jia-qi Liu, Song-nan Wang, Tian-xiao Pang, Qing-feng Wang

**Affiliations:** 1 Liaoning University of Traditional Chinese Medicine, Shenyang, China; 2 Affiliated Hospital of Liaoning University of Traditional Chinese Medicine, Shenyang, China

**Keywords:** cardiometabolic health, dietary intervention, gut microbiota, inflammageing, mediterranean diet, short-chain fatty acids

## Abstract

Rapid global population ageing is accelerating the burden of cardiometabolic disease, and inflammageing—the chronic, low-grade, sterile inflammation that accompanies advancing age—has emerged as a shared pathological substrate linking cardiovascular disease, metabolic syndrome, and type 2 diabetes mellitus in older adults. Increasing evidence identifies the gut microbiota as an upstream driver of this process, forming a modifiable feedback loop with the intestinal barrier, the immune system, and cardiometabolic homeostasis. With advancing age, beneficial taxa such as *Bifidobacterium* and *Faecalibacterium prausnitzii* decline in abundance, whereas pro-inflammatory taxa expand; this dysbiosis compromises tight-junction integrity, producing the “leaky gut” phenotype and permitting translocation of lipopolysaccharide and other pathogen-associated molecular patterns into the portal circulation. The resulting metabolic endotoxaemia activates TLR4/NF-κB signalling and sustains systemic elevations of IL-6, TNF-α, and IL-1β, which converge on insulin resistance, endothelial dysfunction, oxidative stress, and vascular injury. Microbiota-derived metabolites further shape this trajectory: trimethylamine-N-oxide (TMAO) has been implicated in the acceleration of atherosclerosis, foam-cell formation, and platelet reactivity—largely on the basis of rodent studies and observational human data—whereas short-chain fatty acids reinforce barrier function and induce regulatory T cells. This review systematically synthesises current mechanistic and clinical evidence on the gut microbiota–inflammageing axis and its contribution to cardiometabolic deterioration during ageing, and critically appraises dietary intervention strategies as the most direct and actionable entry point into this feedback loop. The Mediterranean, Green-Mediterranean, and plant-based dietary patterns, together with intermittent fasting, have been shown to remodel the gut microbiota, reduce systemic inflammatory tone, and improve cardiometabolic risk profiles. Key dietary components—fibre-derived short-chain fatty acids, polyphenols and their microbial metabolites, omega-3 polyunsaturated fatty acids together with specialised pro-resolving mediators, and age-appropriate protein intake—act through distinct but converging molecular mechanisms to preserve barrier integrity, restore immunometabolic balance, and support healthy vascular ageing. Emerging translational approaches, including precision nutrition guided by individual microbiome profiles, next-generation probiotics, prebiotics and postbiotics, and small-molecule inhibitors targeting TMAO biosynthesis, are also examined, alongside their current translational limitations—including the absence of standardised microbiome analytical pipelines, strain-specific heterogeneity of probiotic effects, and the lack of formal recommendations in current cardiovascular and diabetes guidelines. Safety considerations relevant to frail older adults—such as hypoglycaemia risk during intermittent fasting, bleeding risk with high-dose omega-3 supplementation, and rare bacteraemia associated with probiotic use in immunocompromised individuals—are also highlighted. Research gaps such as scarce long-term follow-up, under-representation of non-Western populations, and heterogeneity across ageing stages are discussed. By integrating mechanistic insight with translational evidence, this review provides a theoretical foundation for the development of individualised nutritional strategies aimed at extending cardiometabolic healthspan in older adults.

## 1 Introduction

Rapid global population ageing continues to escalate the burden of age-related diseases, with cardiovascular disease (CVD) remaining the leading cause of death in older adults. Adults aged 70 years and older bear a disproportionate share of the global CVD burden, and ischaemic heart disease constitutes the primary contributor to both mortality and disability-adjusted life years (DALYs) in this population (1). Updated projections indicate that, between 2025 and 2050, the age-standardised CVD mortality rate is projected to decline by 30.5%, while the crude mortality rate is expected to rise by 73.4% under the demographic pressure of population ageing, with absolute CVD deaths reaching an estimated 35.6 million by 2050 (2). Elucidating the core mechanisms underlying cardiometabolic deterioration in older adults and implementing early intervention have therefore become pivotal priorities for extending healthspan.

Inflammageing refers to the chronic, low-grade, sterile, and systemic inflammatory state that develops with advancing age in the absence of overt infection. It is characterised by sustained mild elevations in circulating pro-inflammatory cytokines—including IL-6, TNF-α, and IL-1β—together with a pro-inflammatory microenvironment shaped by the senescence-associated secretory phenotype (SASP) (3). At the organ-specific level, cardiovascular ageing is governed by eight interconnected molecular hallmarks: disabled macroautophagy, loss of proteostasis, genomic instability, epigenetic alterations, mitochondrial dysfunction, cellular senescence, dysregulated neurohormonal signalling, and inflammation (4). As an integrative node of multiple pathogenic mechanisms, inflammageing is increasingly recognised as a shared pathological substrate underlying cardiovascular disease, metabolic disorders, and cognitive decline (5).

The composition and function of the gut microbiota change substantially with advancing age. Beneficial taxa such as *Bifidobacterium* and *Faecalibacterium prausnitzii* decline in abundance, whereas pro-inflammatory taxa expand; this reconfiguration is closely linked to frailty and adverse health outcomes (6). Mechanistic experiments in rodents have shown that transplanting intestinal contents from aged mice into young germ-free recipients markedly elevates colonic *Il6*, *Tnf*, and *Tlr4* expression and circulating lipopolysaccharide-binding protein (LBP), providing direct in-vivo evidence in this rodent model that the aged microbiota carries immunogenic potential sufficient to drive low-grade inflammation (7); whether this observation generalises to humans remains to be confirmed by carefully controlled clinical studies. Diets rich in fat and sugar weaken tight-junction proteins and compromise intestinal barrier integrity, producing the “leaky gut” phenotype (8). Subsequently, pathogen-associated molecular patterns such as lipopolysaccharide (LPS) translocate via the portal circulation and trigger systemic low-grade inflammation through the TLR4/NF-κB signalling pathway, providing a key conduit linking intestinal barrier dysfunction to age-related diseases (9). The microbiota-derived metabolite trimethylamine-N-oxide (TMAO), produced from the microbial metabolism of choline and carnitine, promotes macrophage foam-cell formation, impairs endothelial function, and enhances platelet reactivity, and has been implicated in the acceleration of atherosclerosis (10).

Cardiometabolic health encompasses three closely interrelated comorbidities: CVD, metabolic syndrome (MetS), and type 2 diabetes mellitus, all of which share insulin resistance, chronic inflammation, and endothelial dysfunction as their core pathological foundations. MetS substantially increases the risk of type 2 diabetes, cardiovascular disease, and all-cause mortality, and is closely associated with multi-system complications (11). Clinical evidence further indicates that individuals with MetS exhibit elevated levels of telomerase activity, DNA/RNA damage, and GDF15 relative to healthy controls, consistent with the hypothesis that metabolic dysregulation accelerates biological ageing (12).

Pharmacological interventions targeting inflammageing show limited efficacy, and dietary strategies acting on the gut microbiota–inflammageing axis have therefore emerged as critical non-pharmacological approaches to preventing cardiometabolic deterioration in older adults. Major cardiovascular and diabetes practice guidelines—including the 2021 ESC Guidelines on Cardiovascular Disease Prevention, the 2023 AHA/ACC Guideline on Chronic Coronary Disease, and the 2024 ADA Standards of Care—consistently endorse Mediterranean, DASH, and plant-forward dietary patterns as first-line lifestyle intervention for cardiometabolic risk reduction in older adults (13–15). A 30-year prospective cohort study reports that long-term adherence to healthy dietary patterns—exemplified by the Alternative Healthy Eating Index (AHEI) and the alternative Mediterranean diet (aMED)—substantially increases the likelihood of preserving cognitive, physical, and mental health at age 70 (16). In a randomised controlled trial conducted in adults with abdominal obesity (DIRECT-PLUS, *n* = 294, 18-month intervention), the Green-Mediterranean diet (n = 294, 18-month intervention) remodelled the gut microbiota, and microbial alterations mediated the diet-induced improvements in body weight and cardiometabolic risk (17). Dietary polyphenols suppress pathobionts, enrich beneficial taxa, and exert cardiometabolic protection through the modulation of key metabolic pathways such as short-chain fatty acid (SCFA) and TMAO biosynthesis (18).

Despite this accumulating evidence, several important gaps limit direct clinical translation: (i) most primary trials focus on middle-aged rather than geriatric cohorts, so the applicability of dietary-microbiota interventions to the oldest-old and to frail older adults remains uncertain; (ii) mechanistic studies are dominated by rodent models, and the extent to which these translate to humans is only beginning to be tested (6); (iii) precision-nutrition strategies show promise but face substantial methodological and reproducibility barriers; and (iv) safety considerations for pharmacological or aggressive dietary interventions in frail older adults have received limited attention in the literature. Against this background, the present review systematically examines the role of the gut microbiota–inflammageing axis in cardiometabolic health among older adults, critically synthesises the latest evidence on dietary interventions that mitigate cardiometabolic deterioration with ageing, explicitly contextualises this evidence within current clinical guidelines and highlights safety considerations relevant to frail older populations, and provides a theoretical foundation for the development of individualised nutritional strategies. The overall structure of the mechanistic pathway is summarised in Figure 1.

**Figure 1:**
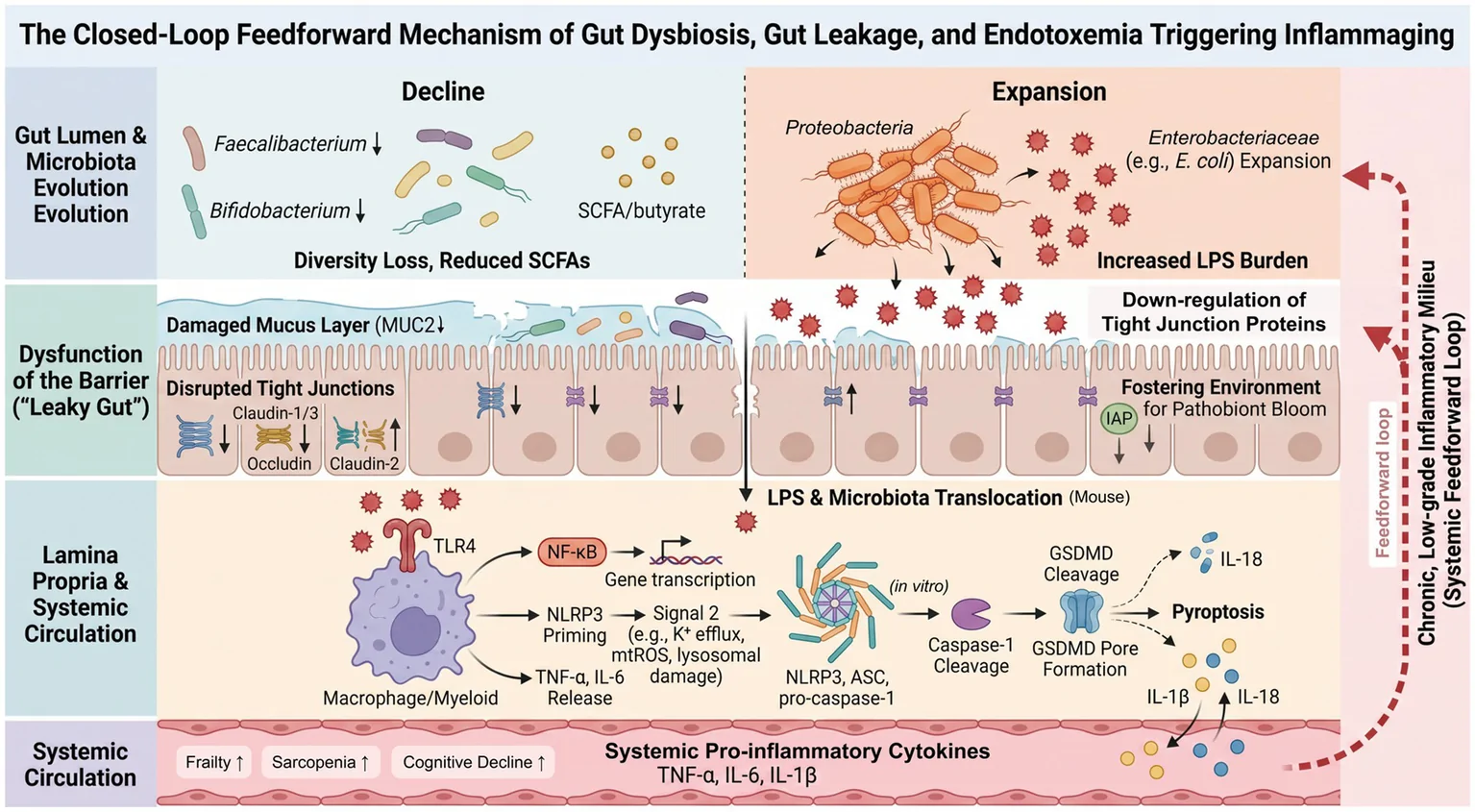
The closed-loop feedforward mechanism of gut dysbiosis, gut leakage, and endotoxaemia triggering inflammageing. Four-tier schematic showing how age-related microbial remodelling can sustain systemic low-grade inflammation. In the gut lumen, beneficial short-chain fatty acid (SCFA)- and butyrate-producing taxa, including *Faecalibacterium* and *Bifidobacterium*, decline, whereas *Proteobacteria* and *Enterobacteriaceae*, including *Escherichia coli*, expand. This shift reduces microbial diversity and increases the luminal lipopolysaccharide (LPS) burden. Barrier dysfunction follows, with thinning of the MUC2 mucus layer, down-regulation of tight-junction proteins, reduced intestinal alkaline phosphatase (IAP) activity and pathobiont expansion. Translocated LPS activates Toll-like receptor 4 (TLR4) and nuclear factor-κB (NF-κB) signalling in macrophages and myeloid cells, inducing tumour necrosis factor-α (TNF-α) and interleukin-6 (IL-6) and priming NLRP3 inflammasome activation. A second signal, including potassium efflux, mitochondrial reactive oxygen species (ROS) and lysosomal damage, promotes NLRP3–ASC–pro-caspase-1 assembly, gasdermin D cleavage, pyroptosis and release of mature interleukin-1β (IL-1β) and interleukin-18 (IL-18). These cytokines feed back to further impair barrier integrity and expand pathobionts, forming a self-reinforcing feedforward loop that contributes to inflammageing.

## 2 Mechanistic interplay between the gut microbiota and inflammageing

Inflammageing is a chronic, low-grade, systemic pro-inflammatory state that develops with advancing age in the absence of overt infection, and it underlies a broad spectrum of age-related diseases (3). The updated framework of the hallmarks of ageing now classifies chronic inflammation and microbial dysbiosis as core constituents of the ageing process (19). The gut microbiota is not a passive bystander but has emerged as an upstream driver of inflammageing (6). Compositional shifts in the microbiota compromise intestinal barrier integrity, allow microbial products to enter the circulation, and persistently activate innate immunity (20). This Section examines the three sequential steps of this process—microbial remodelling, barrier dysfunction, and endotoxin-driven immune activation—and critically evaluates the strength and translational relevance of the underlying evidence.

### 2.1 Age-related remodelling of the gut microbiota

The most robust microbial signature of ageing is declining diversity. Older adults, particularly the frail, exhibit reduced species richness, diminished community stability, and a relative loss of beneficial taxa, including short-chain fatty acid (SCFA) producers (21). This compositional shift parallels rising systemic inflammation (22).

The remodelling, however, is not merely degenerative. Healthy long-lived individuals display a distinctive microbial and metabolic profile, and the degree of compositional uniqueness predicts survival (23). Centenarians are enriched in specific secondary bile acid biosynthetic pathways, consistent with an adaptive component to part of this remodelling (24). Animal experiments in mice further support the notion that the aged microbiota itself amplifies host inflammation: transfer of intestinal contents from aged mice into young germ-free recipients markedly elevates colonic *Il6*, *Tnf*, and *Tlr4* expression together with circulating lipopolysaccharide-binding protein (7). These murine findings await formal replication in controlled human studies.

In parallel with the loss of beneficial taxa, opportunistic pathobionts expand with age. A study of duodenal aspirates from 251 subjects showed that the age-associated decline in small-intestinal diversity is driven primarily by Enterobacteriaceae expansion, with the most pronounced increases in *Escherichia coli* and *Klebsiella* (25). These pathobionts proliferate more readily in an inflammatory milieu and track closely with frailty progression (26).

The central functional consequence of these shifts is impaired butyrate production. Butyrate sustains the intestinal epithelial barrier, drives regulatory T cell differentiation, and suppresses NF-κB signalling (27). Butyrate has recently been characterised as a senomorphic agent: in senescent T cells, it reduces DNA damage, mitochondrial reactive oxygen species, and mTOR activation, and suppresses the release of senescence-associated secretory phenotype factors such as IL-6 (28).

Faecal microbiota transplantation studies in rodents are consistent with a causal role for the aged microbiota in this process: transfer of aged microbiota into young recipients induces ageing-associated phenotypes, whereas transfer of young microbiota partially reverses multiple hallmarks of ageing in the gut, eye, and brain of aged hosts (29). These findings support, but do not by themselves establish for humans, the notion that reduced diversity, depletion of beneficial taxa, and expansion of pathobionts jointly define microbial dysbiosis as a starting point of inflammageing.

Metagenomic surveys have refined this picture at the functional level. Compared with young adults, the intestinal metagenome of older adults shows a systematic contraction of carbohydrate-active enzyme (CAZy) gene families involved in the fermentation of dietary fibre, together with an expansion of gene modules encoding lipopolysaccharide biosynthesis, aromatic amino acid degradation, and mucin-glycan forageing (30). This functional shift means that even where relative taxonomic abundance is only modestly altered, the metabolic output of the ecosystem changes markedly: fibre-derived acetate, propionate and butyrate all decline, while flux through proteolytic and mucin-degrading pathways rises (6). In practical terms, an older person and a younger person consuming the same fibre-containing meal will generate substantially different downstream metabolite profiles, which helps to explain why identical dietary interventions in the elderly frequently produce blunted responses relative to those observed in younger cohorts (31).

Strain-level analyses have added a further layer of complexity. Within individual taxa that persist across the lifespan, such as *Bacteroides fragilis* and several *Bifidobacterium* species, the strains found in older adults often carry attenuated or lost accessory gene clusters, including those encoding polysaccharide utilisation loci and secondary bile acid transformations (32). The taxonomic label is therefore preserved, but the ecological role is not. This phenomenon, sometimes termed ‘functional erosion within a preserved taxon’, cautions against relying on genus- or species-level abundance data alone when characterising the aged microbiome, and highlights the value of metagenomic and metatranscriptomic approaches for capturing the true magnitude of age-related change (21).

Circadian disorganisation is a further, under-appreciated feature of the aged gut ecosystem. Both the composition and the transcriptional activity of gut microbes normally follow diurnal oscillations that are entrained by feeding rhythms and host cortisol signalling (33). In older adults, sleep fragmentation, altered eating schedules, and reduced physical activity blunt these oscillations, and the resulting loss of temporal structure feeds back on the host by desynchronising intestinal clock gene expression, bile acid secretion and immune cell trafficking (34). Restoration of feeding rhythmicity through time-restricted eating has been proposed as one route through which lifestyle can rescue microbial oscillation in older adults (35).

Critical appraisal of the evidence. The picture of an age-associated microbial signature is broadly consistent across cross-sectional cohorts, yet several caveats warrant explicit acknowledgement. First, most large descriptive cohorts (e.g., ELDERMET, TwinsUK, American Gut) are dominated by Western, community-dwelling participants, and non-Western and institutionalised elderly remain under-represented (6). Second, the causal architecture of microbial remodelling and inflammageing in humans rests largely on FMT experiments in rodents, and human interventional data—particularly randomised trials with hard clinical endpoints—remain limited. Third, effect sizes for individual taxa are typically modest, and “universal” microbial biomarkers of ageing have not yet been formally validated across independent cohorts (6). These limitations bear directly on the translational claims discussed in Section 6.

### 2.2 Intestinal barrier dysfunction (the “leaky gut” phenotype)

The intestinal barrier comprises the mucus layer and the epithelial tight junctions, and it constitutes the first line of separation between luminal microbes and the host immune system. Age-related barrier weakening, often termed the “leaky gut” phenotype, is an evolutionarily conserved feature of ageing that spans nematodes, flies, rodents, and primates, and it correlates with systemic health decline and mortality (36).

Mucus barrier deterioration is the initial step. Colonic mucus is built around MUC2, secreted by goblet cells. In the aged colon, the mucus layer becomes more permeable to bacteria, microbes approach the epithelial surface, the goblet-cell proportion declines, *Spdef* expression decreases, Muc2 protein abundance falls, and mucin glycosylation is altered (37).

Tight-junction protein downregulation constitutes the second step. The intercellular seal is formed by occludin, the claudin family, and ZO-1, and it governs paracellular permeability. In the aged and inflamed milieu, sealing claudins (such as claudin-1 and claudin-3), occludin, and ZO-1 are downregulated, whereas the pore-forming claudin-2 is upregulated, leading to barrier “loosening” (38). Rodent studies indicate that the barrier and the microbiota engage in bidirectional regulation: genetic deletion of *claudin-3* simultaneously induces barrier leakage and dysbiosis, and faecal microbiota transplantation transfers colitis susceptibility from claudin-3-deficient mice to germ-free recipients (39).

The immediate consequence of barrier disruption is translocation of microbial products. Intestinal alkaline phosphatase (IAP) detoxifies lipopolysaccharide and helps maintain tight junctions, but its activity declines with age, allowing endotoxin to more readily breach the barrier (40). In aged animal models, restoration of tight-junction protein expression together with microbiota remodelling reverses toxin translocation and systemic inflammation, suggesting that the barrier is a tractable therapeutic node (41).

The regulation of tight-junction integrity is now understood as an actively signalled process rather than a purely structural property. Myosin light-chain kinase (MLCK) is a central effector: its activation by pro-inflammatory cytokines, particularly TNF-α acting through TNFR1, phosphorylates the regulatory light chain of myosin II, triggers perijunctional actomyosin contraction, and induces a rapid, reversible increase in paracellular permeability (42). Sustained MLCK activation, as observed in the aged intestine, produces chronic ‘loosening’ of the epithelium and provides a mechanistic bridge between low-grade systemic inflammation and further barrier failure (43).

The Wnt/β-catenin and Notch signalling pathways govern crypt regeneration and goblet cell differentiation, and both are dysregulated with age. Reduced Wnt tone in the aged crypt base slows intestinal stem cell turnover, prolongs the time required to repair epithelial injury, and shifts the differentiation balance away from goblet cells (44). Concurrently, defective Notch-mediated lateral inhibition further alters the ratio of secretory to absorptive lineages (45). The net consequence is a mucus layer that is not only thinner but also less efficiently replenished after injury, extending the window during which luminal microbes come into close contact with the epithelium (46).

The mucus layer itself is now recognised as a dynamic immune compartment, not merely a physical seal. Goblet-cell-associated antigen passages (GAPs) allow controlled sampling of luminal antigens by lamina propria dendritic cells and support the induction of oral tolerance; GAP formation is regulated by acetylcholine acting through muscarinic M4 receptors and is impaired in aged mice, correlating in preclinical studies with a loss of tolerogenic dendritic cell phenotypes (47). These findings reframe the ageing barrier as a coordinated failure of physical, secretory, and immunological functions, rather than a simple loss of mechanical containment (48).

Emerging evidence also implicates cellular senescence in the epithelium itself. Senescent enterocytes and Paneth cells accumulate with age, adopt a senescence-associated secretory phenotype (SASP), and secrete IL-6, IL-8 and matrix metalloproteinases that further disrupt junctional integrity and reshape the local mucosal niche (49). Selective clearance of senescent cells using senolytic combinations, such as dasatinib plus quercetin, has been reported in preclinical models to improve intestinal barrier indices and reduce circulating markers of endotoxaemia, offering a conceptual complement to microbiota- and diet-based approaches (50). Whether senolytic strategies improve barrier function in older humans has yet to be established in randomised trials.

Critical appraisal of the evidence. The mechanistic hierarchy from mucus thinning through tight-junction remodelling to systemic endotoxaemia is well characterised in rodent models, but three limitations apply when transposing this framework to older adults. First, non-invasive biomarkers of human intestinal permeability—including lactulose/mannitol ratio, plasma zonulin, and LBP—correlate imperfectly with each other and with histological barrier measures, so the clinical operational definition of a “leaky gut” remains contested. Second, published human studies vary widely in age range, comorbidity burden, and medication exposure (particularly proton-pump inhibitors and NSAIDs, both of which independently affect barrier function), limiting cross-study comparability. Third, no intervention targeting a single barrier component has yet demonstrated a hard clinical benefit in older adults in an adequately powered randomised trial (51). The barrier therefore remains an attractive but not yet validated therapeutic node.

### 2.3 Endotoxaemia and immune activation

Lipopolysaccharide (LPS), a component of the Gram-negative bacterial outer membrane, is the central molecule linking barrier leakage to systemic inflammation. After barrier disruption, plasma LPS rises mildly but persistently, producing the state termed metabolic endotoxaemia; LPS is presented through LPS-binding protein, CD14, and MD-2 to Toll-like receptor 4 (TLR4) on myeloid cells, which activates NF-κB and drives transcription of pro-inflammatory cytokines including TNF-α and IL-6 (52).

The TLR4 signal also upregulates NLRP3 and pro-IL-1β at the transcriptional level, delivering the first signal for inflammasome activation; potassium efflux, mitochondrial reactive oxygen species, and lysosomal damage then provide the second signal that drives oligomerisation of NLRP3, ASC, and pro-caspase-1; activated caspase-1 cleaves pro-IL-1β and pro-IL-18 into their mature forms and cleaves gasdermin D to execute pyroptosis (53). The NLRP3 inflammasome is therefore increasingly regarded as a central node integrating ageing-associated danger signals, and its chronic activation is closely linked to age-related disorders including metabolic diseases, cardiovascular diseases, and neurodegenerative conditions (54).

A causal role for this pathway has been demonstrated in a rat model of atrial fibrillation: the aged microbiota activates atrial NLRP3 through elevated LPS and glucose and promotes age-related atrial fibrillation; transfer of the aged microbiota into young hosts transmits this susceptibility (55). Whether analogous mechanisms operate in humans remains to be tested in clinical cohorts.

At the clinical level, serum IL-6 is consistently elevated in older adults with comorbidities relative to healthy controls and provides a quantifiable index of peripheral low-grade inflammation (56). Preliminary evidence from small, short-duration dietary intervention trials further indicates that probiotic supplementation reduces CRP and IL-6 levels in older adults with frailty or sarcopenia, suggesting that this axis is amenable to nutritional modulation; however, current evidence remains heterogeneous with respect to probiotic strain, dose, and duration, and larger trials with hard clinical endpoints are needed before firm recommendations can be made (57).

A self-sustaining feed-forward circuit therefore emerges: LPS translocation → TLR4/NF-κB activation → NLRP3 inflammasome assembly → IL-1β/IL-18 release and pyroptotic amplification. The pro-inflammatory cytokines produced by this circuit, in turn, suppress tight-junction proteins and shape a niche that favours pathobiont expansion, returning the system to the dysbiotic starting point described in Section 2.1 (55). This closed loop accounts for the reciprocal causation among the microbiota, the barrier, and innate immunity during ageing, and it defines clear targets for anti-ageing interventions that modulate the microbiota, restore barrier integrity, or block the TLR4–NLRP3 axis.

Beyond the canonical TLR4–NLRP3 axis, several parallel innate immune sensors are now recognised as contributors to inflammageing. AIM2, an inflammasome sensor that recognises cytosolic double-stranded DNA, is activated when microbial DNA translocates across a compromised barrier, and its activation triggers a caspase-1-dependent programme convergent with that of NLRP3 (58). Non-canonical inflammasome signalling through caspase-11 (or caspase-4/5 in humans), which detects cytosolic LPS directly, provides a further route by which barrier leakage engages inflammatory responses independently of surface TLR4 (59). In aged tissues, the simultaneous activation of these convergent sensors amplifies the inflammatory output beyond what any single blockade can attenuate, which is consistent with the modest effect sizes reported for interventions targeting NLRP3 alone (54).

The cGAS–STING pathway represents another link between microbial exposure and inflammageing. Cytosolic DNA, whether of microbial or mitochondrial origin, is sensed by cyclic GMP–AMP synthase (cGAS), which generates the second messenger 2′3′-cGAMP and activates the stimulator of interferon genes (STING) (60). STING drives type I interferon responses and, in the ageing context, sustains a chronic interferon signature that is now recognised as a component of the SASP (61). Because mitochondrial dysfunction in senescent cells releases mitochondrial DNA into the cytosol, and because bacterial DNA leaking from a permeable barrier further engages cGAS, the pathway sits at a confluence of microbial and mitochondrial cues that are both amplified with age (62).

Immune ageing itself modifies the cellular substrate on which these signals act. Myeloid-biased haematopoiesis, expansion of tissue-resident macrophages with a pro-inflammatory transcriptome, and clonal haematopoiesis of indeterminate potential (CHIP)—particularly clones bearing mutations in *TET2* or *DNMT3A*—each lower the threshold for inflammasome activation and heighten cytokine output in response to endotoxin (63). In older adults, therefore, the same absolute dose of translocated LPS provokes a larger and more sustained systemic response than it would in a younger host (64). This age-dependent amplification provides one explanation for the observation that interventions restoring barrier integrity, even modestly, tend to produce disproportionately large reductions in circulating inflammatory markers in the elderly (57) (see Figure 1; Table 1).

**Table 1:** Age-related changes across the gut microbiota–intestinal barrier–innate immunity axis and their molecular consequences.

Compartment	Age-related change	Molecular/Functional consequence	Refs
Gut microbiota composition	↓ *Faecalibacterium prausnitzii*, *Bifidobacterium* spp., *Roseburia*; ↑ Proteobacteria and *Enterobacteriaceae* (e.g., *E. coli*)	Loss of α-diversity; reduced short-chain fatty acid (SCFA) output; expansion of LPS-producing Gram-negative organisms	(6, 7)
Mucus layer	MUC2 secretion ↓; goblet-cell proportion ↓; S*pdef* expression ↓; altered mucin glycosylation	Thinner, more permeable inner mucus layer; commensals approach epithelium; loss of tolerogenic mucosal environment	(37, 46–48)
Tight-junction proteins	↓ Claudin-1/3, occludin, ZO-1; ↑ pore-forming claudin-2; sustained MLCK activation	Paracellular permeability ↑ (“leaky gut”); chronic actomyosin contraction of the perijunctional ring	(38, 39, 42, 43)
Detoxification enzymes	↓ Intestinal alkaline phosphatase (IAP) activity	Reduced LPS dephosphorylation; increased LPS bioactivity at the epithelial interface	(40)
Epithelial regeneration	↓ Wnt/β-catenin tone in crypt base; defective Notch lateral inhibition	Slower stem-cell turnover; secretory-to-absorptive lineage imbalance; delayed post-injury repair	(44–46)
Epithelial senescence	Accumulation of senescent enterocytes and Paneth cells with SASP	Local secretion of IL-6, IL-8 and MMPs; junctional disassembly; senolytic (dasatinib + quercetin) reversibility in preclinical models	(49, 50)
Innate immune sensing	TLR4/NF-κB priming; NLRP3, AIM2 and non-canonical caspase-11 activation; cGAS–STING engagement	Elevated TNF-α, IL-6, IL-1β and IL-18; pyroptosis via GSDMD cleavage; type I interferon signature	(52–56, 58–62)
Systemic immune ageing	Myeloid-biased haematopoiesis; CHIP (TET2, DNMT3A); expansion of pro-inflammatory tissue macrophages	Lower threshold for inflammasome activation; disproportionate cytokine response to a given LPS dose	(63, 64)

Clinical translation and biomarker readiness. Although the mechanistic model outlined above is well established, no single biomarker of endotoxaemia has been formally endorsed by current cardiovascular or diabetes guidelines. Circulating LPS is technically difficult to measure reproducibly and is confounded by acute-phase changes; LBP and soluble CD14 are more stable proxies but lack validated clinical thresholds in older adults. IL-6 and hs-CRP remain the most widely applicable markers of low-grade inflammation in geriatric practice and are the readouts most commonly used in intervention trials. Clinically actionable endotoxaemia biomarkers in older adults therefore represent an unmet need that will need to be addressed before mechanism-based interventions can be routinely deployed (51).

## 3 Cardiometabolic disorders: the link between metabolic syndrome and diabetes

Metabolic syndrome (MetS) and type 2 diabetes mellitus (T2DM) represent central nodes in the cardiometabolic injury associated with ageing. They share common pathological foundations, including insulin resistance, low-grade inflammation, and lipid dysregulation (65). Recent studies have identified the gut microbiota as a common upstream regulator of this process (66). Within the conceptual framework of the “hallmarks of ageing”, microbial dysbiosis and chronic inflammation are listed as core hallmarks that are intricately intertwined with metabolic dysregulation (19, 67). Building on the microbiota–barrier–immunity axis introduced in Section 2, the present Section extends the narrative downstream, illustrating how dysbiosis progresses through metabolic syndrome to diabetes and ultimately culminates in cardiovascular injury (Figure 2).

**Figure 2:**
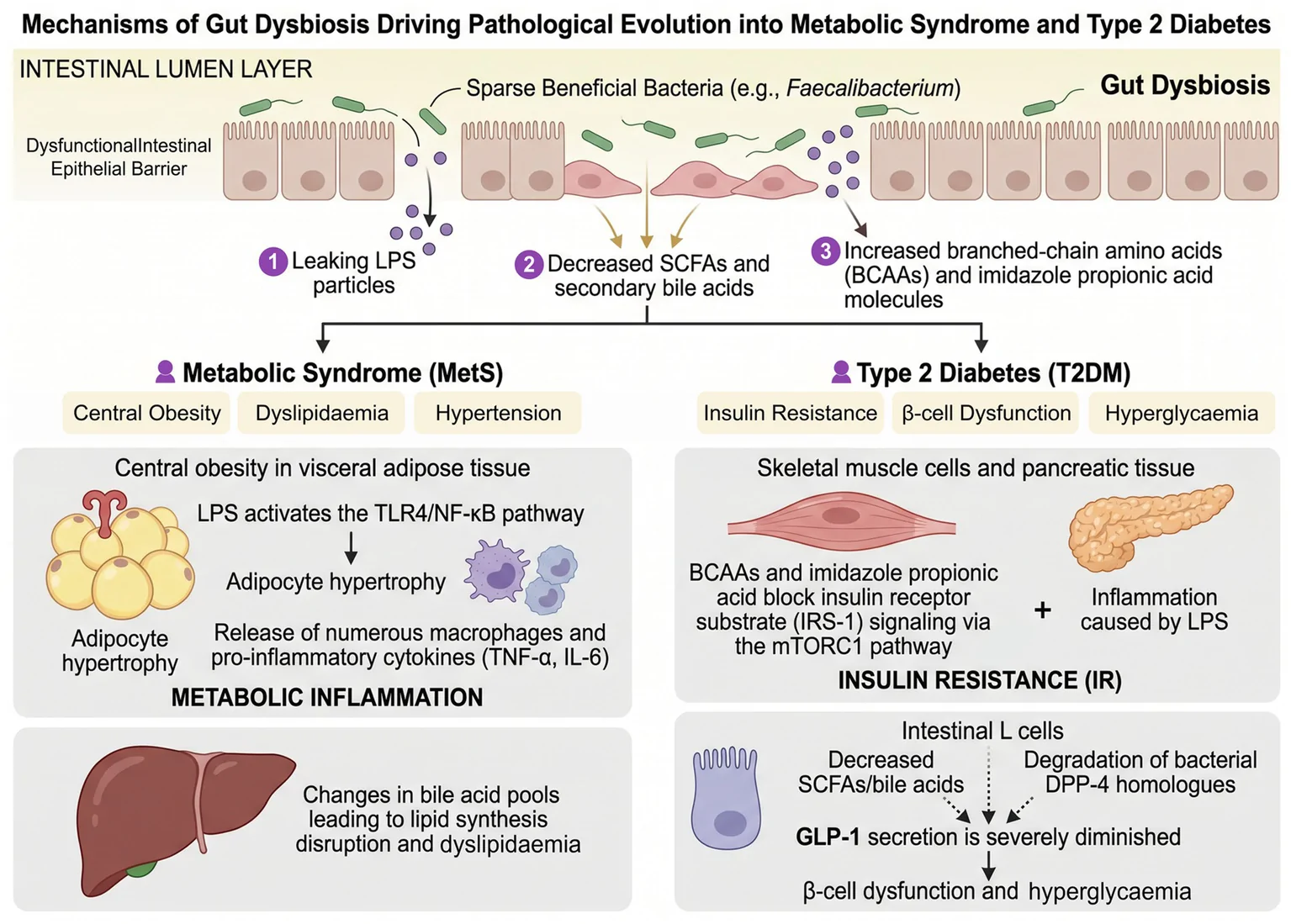
Mechanisms of gut dysbiosis driving pathological evolution into metabolic syndrome and type 2 diabetes. Schematic of three microbiota-derived signals that translate age-related dysbiosis into Metabolic syndrome (MetS) and Type 2 diabetes mellitus (T2DM). A dysfunctional intestinal epithelial barrier with sparse beneficial bacteria, including *Faecalibacterium*, permits LPS leakage, reduces SCFA and secondary bile acid production, and increases branched-chain amino acids (BCAAs) and Imidazole propionate. In the MetS panel, LPS activates TLR4/NF-κB signalling in visceral adipose tissue, promoting adipocyte hypertrophy and macrophage recruitment, with release of TNF-α and IL-6. Altered bile acid metabolism further disrupts hepatic lipid handling, contributing to dyslipidaemia, central obesity and hypertension. In the T2DM panel, BCAAs and Imidazole propionate impair insulin receptor substrate-1 (IRS-1) signalling through the mechanistic target of rapamycin complex 1 (mTORC1) pathway, while LPS-mediated inflammation amplifies insulin resistance. Reduced SCFAs and bile acids, together with bacterial dipeptidyl peptidase-4 (DPP-4)-like activity, impair glucagon-like peptide-1 (GLP-1) signalling, β-cell function and glycaemic control.

### 3.1 The central role of metabolic syndrome in ageing

Metabolic syndrome is characterised by central obesity, dyslipidaemia, hyperglycaemia, and hypertension. Its prevalence rises markedly with advancing age, making it a critical cluster of cardiovascular risk factors in the elderly population (66). The gut microbiota participates extensively in this process by modulating energy harvest, adipose tissue function, and immune responses (68).

Central (visceral) obesity is a core component of metabolic syndrome. Bidirectional crosstalk exists between the gut microbiota and adipose tissue, and dysbiosis is associated with visceral fat accumulation and adipose tissue inflammation (69). In individuals with obesity, the ratio of Firmicutes to Bacteroidetes (F/B ratio) is commonly elevated, although the reliability of this index as a biomarker remains debated—recent large-cohort re-analyses show that the F/B ratio varies considerably across populations, dietary contexts, and sequencing pipelines, and its diagnostic value for obesity has been questioned (70). More robust changes include a decline in short-chain fatty acid (SCFA)-producing beneficial bacteria and a reduction in microbial diversity (71, 72).

Lipid dysregulation is also closely linked to the microbiota. The gut microbiota influences host lipid profiles by modulating bile acid metabolism and cholesterol pathways (73). Microbiota-derived metabolites alter hepatic lipid synthesis and lipoprotein processing, contributing to dyslipidaemia; this process, together with changes in energy-harvest efficiency, has been proposed to jointly drive the progression of obesity (74).

Microbiota-derived pro-inflammatory metabolites constitute a key link between metabolic syndrome and ageing. Upon barrier disruption, lipopolysaccharide (LPS) enters the systemic circulation, producing a sustained low-grade state termed “metabolic endotoxaemia” (52). Circulating LPS activates NF-κB via TLR4, induces pro-inflammatory cytokines, and drives low-grade systemic inflammation (52). Concurrently, anti-inflammatory metabolites such as SCFAs are diminished in the dysbiotic state, leaving pro-inflammatory signals unopposed (75). This microbiota-driven “metabolic inflammation” has been proposed as a common pathological basis for obesity, diabetes, and fatty liver disease (69), with a mutually reinforcing relationship existing among obesity, dysbiosis, and inflammation (76). This low-grade inflammatory state overlaps substantially with the ageing-associated state of “inflammageing,” and the two appear to synergistically accelerate the functional decline of metabolic organs (19, 67).

### 3.2 Gut microbiota and type 2 diabetes mellitus

Type 2 diabetes mellitus is pathologically defined by insulin resistance (IR) and β-cell dysfunction. Accumulating evidence indicates that gut dysbiosis is actively involved in this pathogenic process (66). The microbiota of patients with diabetes is typically characterised by a reduction in butyrate-producing bacteria, an expansion of opportunistic pathogens, and a decrease in overall diversity (77); higher microbial α-diversity is associated with lower levels of insulin resistance (77). These associations are cross-sectional, and causal directionality in humans requires further prospective validation.

#### 3.2.1 Dysbiosis-mediated insulin resistance

The microbiota can impair insulin sensitivity through multiple pathways. The first is metabolic endotoxaemia, as described above: barrier leakage allows LPS to enter the circulation, where TLR4-driven low-grade inflammation interferes with insulin signalling and promotes insulin resistance (78). This tripartite “microbiota–immunity–glucose metabolism” interaction is regarded as a core mechanism underlying low-grade inflammation in diabetes (78). The second is the action of specific microbial metabolites: branched-chain amino acids (BCAAs) and imidazole propionate suppress insulin signalling through the mTORC1 pathway, thereby impairing glucose handling (79). These mechanistic findings elevate microbiota-derived metabolites from mere correlates to plausible causal contributors to host metabolism, although human interventional confirmation is still limited (79). The third is the loss of protective metabolites: SCFAs improve insulin sensitivity in peripheral tissues and suppress inflammation, and their reduction may exacerbate metabolic imbalance (75); alterations in the bile acid pool similarly attenuate TGR5 and FXR signalling, further damageing the intestinal barrier and aggravating hepatic insulin resistance (80).

#### 3.2.2 Attenuation of incretin secretion

Incretins are important regulators of glucose metabolism. SCFAs act on GPR43 receptors on intestinal L cells to promote the secretion of glucagon-like peptide-1 (GLP-1), and bile acids stimulate GLP-1 release via TGR5 (80, 81). Dysbiosis reduces SCFAs and secondary bile acids, thereby attenuating GLP-1 secretion (81). In addition, certain gut bacteria express homologues of human dipeptidyl peptidase-4 (DPP-4), which can directly degrade GLP-1 and diminish its bioactivity (80). These mechanisms collectively contribute to insufficient postprandial insulin secretion and sustained hyperglycaemia. Notably, certain beneficial bacteria can partially reverse this impairment—for example, *Akkermansia muciniphila* exhibits GLP-1 agonist-like properties, lowering hyperglycaemia and reinforcing barrier function in preclinical and early clinical studies (81, 82). In summary, gut dysbiosis may drive the onset and progression of T2DM through two complementary routes: aggravating insulin resistance and attenuating incretin secretion (83) (Figure 2).

Clinical translation. Current diabetes practice guidelines (ADA Standards of Care 2024, IDF 2025 clinical practice recommendations) acknowledge the emerging role of the gut microbiota in T2DM pathogenesis but do not yet recommend routine microbiota profiling for diagnosis, risk stratification, or treatment selection (15). Meta-analyses of probiotic and prebiotic trials in T2DM report small-to-moderate improvements in fasting glucose, HbA1c, and HOMA-IR (weighted mean HbA1c reductions of roughly 0.2–0.4%), yet effects on hard endpoints—cardiovascular events, incident T2DM, all-cause mortality—have not been demonstrated (see Section 6.2). Microbiota-targeted strategies should therefore currently be viewed as adjuncts to, rather than replacements for, guideline-directed pharmacotherapy in older adults with T2DM.

### 3.3 The cascade toward cardiovascular injury

Metabolic syndrome and diabetes ultimately converge on cardiovascular injury. Atherosclerosis is a chronic inflammatory vascular disease whose pathogenesis involves lipid dysregulation, inflammation, oxidative stress, and endothelial dysfunction (84); the gut microbiota has been implicated as a contributor to this process through multiple pathways (85) (Figure 3).

**Figure 3:**
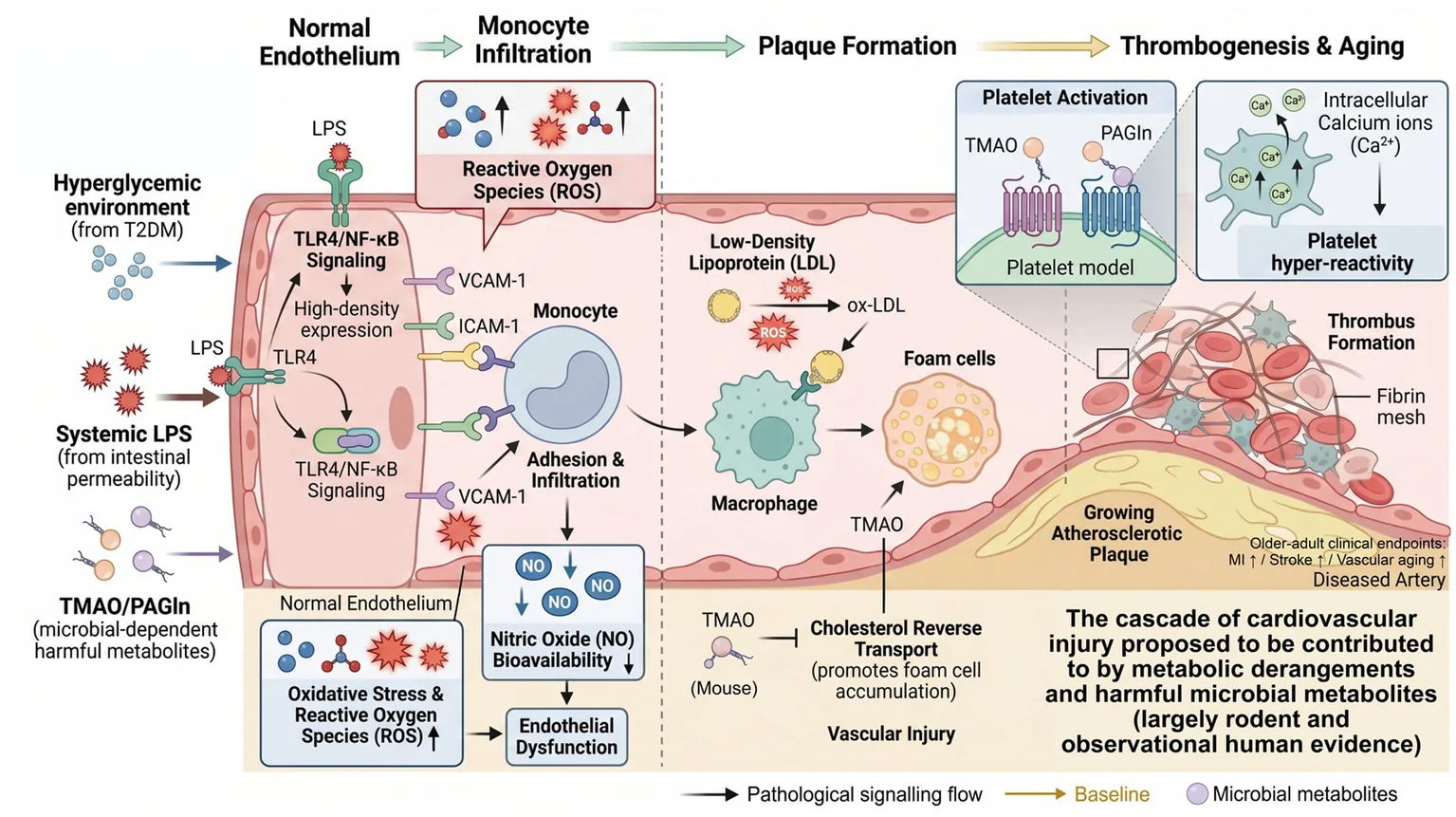
The cascade of cardiovascular injury orchestrated by metabolic derangements and harmful microbial metabolites. Schematic overview of how hyperglycaemia, systemic LPS derived from a permeable intestinal barrier, and microbial metabolites, including trimethylamine-N-oxide (TMAO) and phenylacetylglutamine (PAGln), converge on endothelial injury, plaque formation and thrombogenesis. LPS activates TLR4/NF-κB signalling in endothelial and immune cells, up-regulating vascular cell adhesion molecule-1 (VCAM-1) and intercellular adhesion molecule-1 (ICAM-1), which promote monocyte adhesion and infiltration. ROS accumulation oxidises low-density lipoprotein (LDL) to ox-LDL, driving macrophage uptake and foam-cell formation. TMAO is proposed to inhibit reverse cholesterol transport and thereby support foam-cell accumulation, largely on the basis of rodent and observational human evidence. Reduced nitric oxide (NO) bioavailability, together with oxidative stress, promotes endothelial dysfunction. TMAO and PAGln also enhance platelet reactivity, increase intracellular calcium signalling and support fibrin-rich thrombus formation on growing atherosclerotic plaques, linking gut microbial metabolism to accelerated vascular ageing.

#### 3.3.1 Inflammation, oxidative stress, and accelerated atherosclerosis

LPS released through barrier leakage upregulates vascular cell adhesion molecule-1 (VCAM-1) and intercellular adhesion molecule-1 (ICAM-1) via the TLR4/NF-κB pathway, thereby promoting monocyte adhesion and infiltration (85). Infiltrating monocytes differentiate into macrophages that engulf oxidised lipids to form foam cells, the cellular core of atherosclerotic plaques (86). Concurrently, dysbiosis-associated metabolites induce reactive oxygen species (ROS) production in endothelial and immune cells, while the depletion of protective SCFAs weakens antioxidant defences (87). Excessive ROS damages the endothelial barrier and activates the NF-κB pathway and the NLRP3 inflammasome, generating a positive feedback loop in which oxidative stress and inflammation mutually amplify each other (55). Oxidative stress thus acts as a shared downstream effector linking metabolic dysregulation to vascular injury (88). In the context of ageing, these mechanisms layer upon pre-existing inflammageing, contributing to the early onset of endothelial dysfunction (89).

#### 3.3.2 Pro-thrombotic actions of key metabolites such as TMAO

Trimethylamine N-oxide (TMAO) is a microbiota-dependent pro-atherogenic metabolite (90). Dietary choline and L-carnitine are metabolised by gut bacteria into trimethylamine (TMA), which is subsequently oxidised to TMAO in the liver by flavin-containing monooxygenase 3 (FMO3) (90). TMAO has been reported to aggravate atherosclerotic plaques by promoting foam cell formation, impairing reverse cholesterol transport, and inducing endothelial dysfunction (84, 90). Notably, TMAO also exerts pro-thrombotic effects: it enhances platelet reactivity to stimuli and promotes intracellular calcium release, thereby increasing the risk of platelet aggregation and thrombus formation (85). Another microbiota-derived metabolite, phenylacetylglutamine (PAGln), similarly enhances platelet reactivity via adrenergic receptors, suggesting that pro-thrombotic effects may be a generalisable feature of microbial metabolites (91). These mechanisms make microbial metabolites attractive targets for cardiovascular risk intervention (82, 92).

The role of TMAO is particularly prominent in ageing-related vascular injury. Circulating TMAO levels increase with advancing age (93); in healthy middle-aged and older humans and in mice, elevated TMAO is associated with vascular oxidative stress and endothelial dysfunction, and impairs vasodilation by suppressing nitric oxide bioavailability (88). TMAO further induces aortic stiffening and raises systolic blood pressure in aged mice, and in the same rodent model pharmacological or dietary suppression of TMAO production has been reported to partially restore endothelium-dependent vasodilation and reduce arterial stiffness toward levels observed in young controls (89). In humans, however, current evidence linking TMAO to vascular ageing remains largely observational, and no interventional trial has yet demonstrated that lowering circulating TMAO reverses vascular ageing in older adults (93, 94). Taken together, these findings—derived largely from rodent studies and observational human data—link microbial metabolites to vascular ageing (93, 94). Moreover, dysbiosis-associated LPS together with hyperglycaemia can promote age-related cardiovascular events such as atrial fibrillation through activation of the NLRP3 inflammasome (55).

TMAO as a clinical biomarker. Despite mechanistic plausibility, TMAO is not currently included in any major cardiovascular risk stratification guideline (ESC 2021, AHA/ACC 2019 primary-prevention guideline, 2023 chronic coronary disease guideline). Meta-analyses and umbrella reviews report dose–response associations between circulating TMAO and adverse cardiovascular outcomes, but heterogeneity across assay platforms (LC–MS versus enzymatic), reference ranges, and fasting-state definitions has limited clinical uptake. Prospective validation in older, geriatric-representative cohorts and standardisation of measurement methodology are prerequisites for translation (95).

In summary, gut dysbiosis, via metabolic syndrome and diabetes, progressively injures the cardiovascular system through a cascade of inflammation, oxidative stress, atherosclerosis, and thrombosis (96). This sequence dovetails with the microbiota–barrier–immunity axis described in Section 2 to form a coherent chain running through inflammageing, and it supports the view that targeting the gut microbiota and its metabolites represents a promising interventional avenue for delaying cardiometabolic ageing. The clinical and translational implementation of this framework—including the current guideline landscape, safety considerations in frail older adults, and the strength of existing intervention trials—is examined in Section 6.

## 4 Dietary patterns as modulators of the axis

Diet is the primary environmental factor that shapes the gut microbiota, influencing its composition and function across the life course (74). Dietary components are converted by the microbiota into a range of metabolites that feed back on the host barrier, immunity and metabolism (80). Diet therefore acts as a key lever for intervening in the microbiota–barrier–immunity–cardiometabolic axis. Against the background of ageing, dietary patterns can accelerate or slow inflammageing (19, 21, 67). This Section discusses the pro-inflammatory Western diet, the protective Mediterranean diet, and two emerging patterns—plant-based diets and intermittent fasting—with critical attention to the strength of the underlying evidence and to safety considerations relevant to older adults.

### 4.1 A pro-inflammatory pattern: the Western diet

The Western diet is characterised by high saturated fat, refined sugars, high salt and low fibre intake (97). It appears to worsen the gut environment through several routes and accelerates inflammageing.

High saturated fat weakens the epithelial barrier through microbiota-mediated effects and increases intestinal permeability, allowing translocation of bacterial metabolites into the circulation (97). Low fibre intake deprives short-chain fatty acid (SCFA)-producing bacteria of their fermentation substrate. The combined result is a reduction in beneficial taxa, expansion of endotoxin-producing Gram-negative organisms such as *Escherichia coli*, and a decline in anti-inflammatory SCFA output (75).

High-fat meals raise postprandial plasma lipopolysaccharide (LPS) levels (97). Barrier leakage and diet-derived LPS together drive endotoxin entry into the systemic circulation, where LPS activates NF-κB via TLR4 and induces pro-inflammatory cytokines, producing a state of low-grade systemic inflammation (52). This diet-driven “metabolic inflammation” has been proposed as a shared pathological basis for obesity, type 2 diabetes and fatty liver disease (69).

The Western diet is rich in red meat and high-fat foods, which provide choline and L-carnitine as substrates for trimethylamine N-oxide (TMAO) production (90). Elevated TMAO has been implicated in the promotion of atherosclerosis and thrombosis (90).

These changes overlap with age-associated inflammageing. Long-term adherence to the Western pattern is associated with aggravated dysbiosis, barrier leakage and endotoxaemia, sustains low-grade inflammation, and appears to accelerate the functional decline of metabolic organs (68). In older populations, long-term adherence to the Western pattern is an important modifiable contributor to inflammageing (67) (Figure 4).

**Figure 4:**
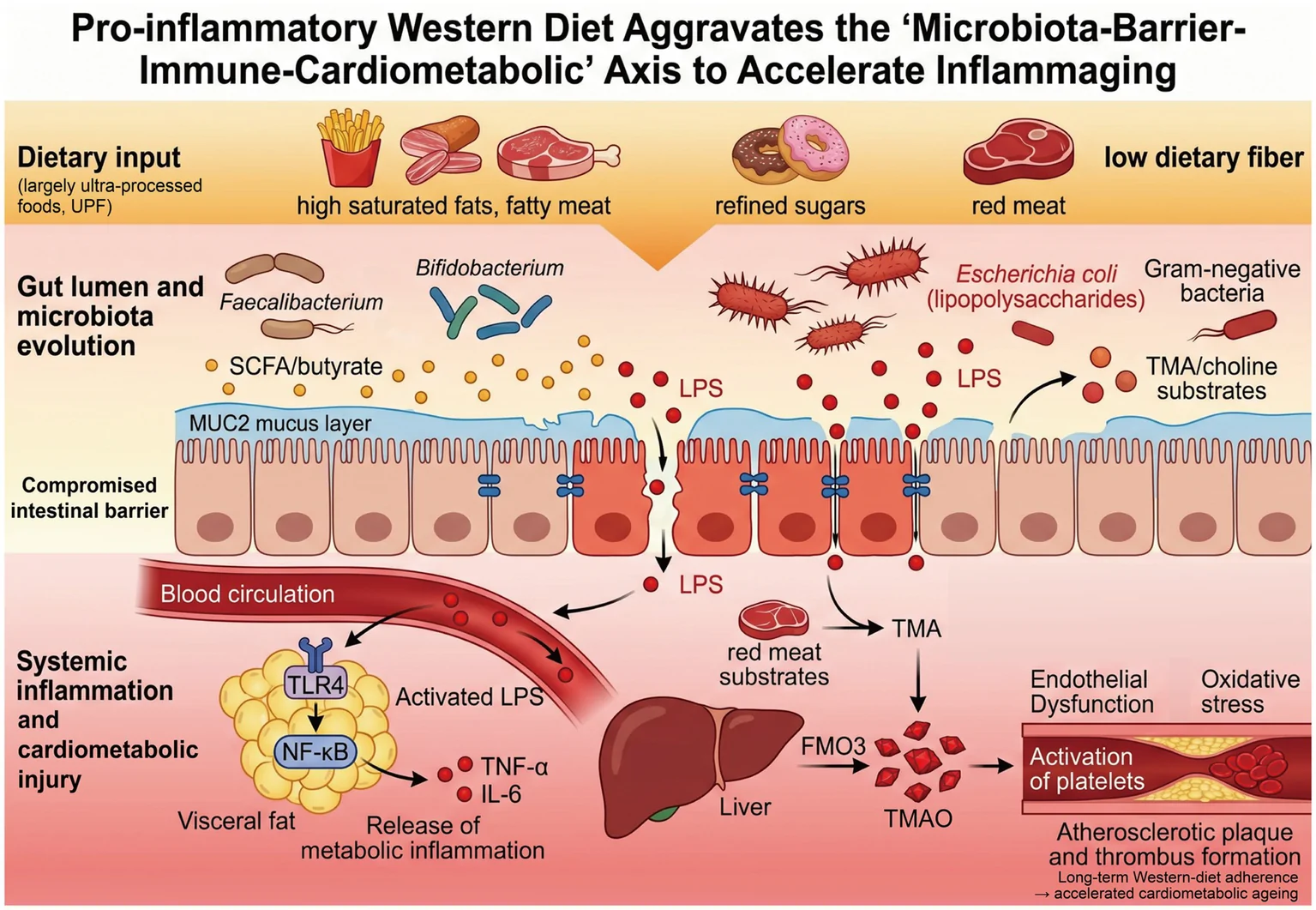
The pro-inflammatory Western diet aggravates the “microbiota–barrier–immune–cardiometabolic” axis to accelerate inflammageing. Integrated schematic showing how a Western dietary pattern, characterised by high saturated fat, fatty meat, refined sugar, red meat and ultra-processed foods, together with low dietary fibre intake, may propagate cardiometabolic injury through four connected compartments. In the gut lumen, fibre deprivation reduces SCFA and butyrate production by beneficial taxa such as *Faecalibacterium* and *Bifidobacterium*, while Gram-negative bacteria, including *Escherichia coli*, expand and increase the LPS and trimethylamine (TMA) substrate pool. At the intestinal barrier, MUC2 mucus depletion and tight-junction disruption produce a compromised intestinal barrier that permits paracellular LPS translocation. In the systemic compartment, LPS activates TLR4/NF-κB signalling in visceral adipose tissue, driving TNF-α and IL-6 release and metabolic inflammation. In parallel, red-meat-derived choline and carnitine are converted by gut microbes to TMA, which is oxidised by hepatic flavin-containing monooxygenase 3 (FMO3) to TMAO.

Critical appraisal of the evidence. The causal contribution of the Western dietary pattern to inflammageing in older adults rests largely on cross-sectional cohorts, mechanistic rodent studies, and short-duration crossover feeding trials in metabolically healthy adults, rather than on long-term randomised trials in geriatric populations. The composite “Western diet” label also aggregates food groups (refined sugars, saturated fat, ultra-processed foods, low fibre) whose individual contributions are difficult to disentangle. Recent large cohort re-analyses using ultra-processed food (UPF) scores have offered more granular exposure metrics and have consistently linked higher UPF intake to increased cardiometabolic risk, but reverse causation and residual confounding cannot be fully excluded (98). The direction and consistency of the evidence, however, support avoidance of a Western-style eating pattern as a defensible clinical recommendation in older adults, even in the absence of dedicated geriatric RCTs.

### 4.2 An anti-inflammatory/protective pattern: the Mediterranean diet

The Mediterranean diet is high in fibre, monounsaturated fatty acids (MUFAs) and polyphenols, and is regarded as a dietary template for healthy ageing (31). The NU-AGE study conducted a one-year randomised intervention in older adults across five European countries; adherence to the Mediterranean diet enriched anti-inflammatory and SCFA-producing taxa such as *Faecalibacterium prausnitzii* and *Roseburia*, while reducing taxa associated with age-related diseases (31). The microbial changes correlated with improved frailty indices, better cognitive function, and lower levels of inflammatory markers including C-reactive protein and IL-17 (31).

Dietary fibre is central to this protective effect. Fibre is the principal substrate for gut microbial fermentation, and its SCFA products maintain barrier integrity, induce regulatory T cells, and suppress inflammation (27). Fibre-targeted dietary interventions have been shown to modulate human immune status (99). MUFAs (mainly from olive oil) and polyphenols add further anti-inflammatory effects. Polyphenols enrich *Bifidobacterium* and *Lactobacillus*, and their microbial metabolites exert anti-inflammatory and cardiovascular protective activity (100).

The metabolic benefits of the Mediterranean diet are closely linked to microbial changes. In overweight and obese subjects, the diet lowered plasma cholesterol and modified the gut microbiome and metabolome independently of energy intake (101). A randomised controlled trial of a Green-Mediterranean diet (DIRECT-PLUS, *n* = 294, 18-month intervention) found that its cardiometabolic benefits were linked to specific microbial shifts (17). In the Health Professionals Follow-Up Study cohort, the protective effect of the Mediterranean diet on cardiometabolic risk was partly mediated by the gut microbiota and was more pronounced in individuals with lower abundance of *Prevotella copri* (102). Meta-omics studies support the link between diet, microbiota and cardiometabolic health (103). Individual microbiota composition predicts cardiometabolic markers and modulates dietary response, supporting a personalised dimension of the diet–microbiota interaction (104).

Critical appraisal of the evidence. Existing evidence for a Mediterranean pattern deserves nuanced appraisal. PREDIMED and its extensions provide the most robust primary-prevention data for a Mediterranean diet on hard cardiovascular endpoints, but the trial has been through a well-publicised data-integrity re-analysis (2018 revision of the 2013 report), and even the corrected effect estimates rest on a modest number of incident events in the highest-risk stratum. DIRECT-PLUS elegantly couples microbiota shifts to cardiometabolic improvement, but has a modest sample (*n* = 294), a follow-up of 18 months, and a predominantly male, workplace-recruited cohort (17). Cohort evidence in older adults is largely observational and susceptible to residual confounding by socio-economic status and overall lifestyle. Head-to-head comparisons across dietary patterns remain scarce, and standardised operational definitions of “Mediterranean adherence” are lacking. Overall the evidence supporting the Mediterranean pattern is strong in middle-aged adults with moderate cardiovascular risk, but of lower certainty for the oldest-old, in whom dedicated trials are still needed (105). This pattern is nevertheless a Class I recommendation in the 2021 ESC Cardiovascular Disease Prevention Guidelines and is endorsed by the 2024 ADA Standards of Care as a first-line dietary approach in T2DM.

### 4.3 Emerging patterns: plant-based diets and intermittent fasting

Plant-based diets. Vegetarian and vegan diets are rich in fibre and polyphenols and promote a more diverse microbial structure (106). A systematic review reported that plant-based diets increase SCFA-producing taxa, raise SCFA levels, and lower inflammatory markers (106). A prospective cohort study found that long-term adherence to plant-based dietary patterns was associated with favourable microbial signatures and better cardiometabolic markers (107). A large study of more than 20,000 individuals showed that the microbial signatures of vegetarian and vegan diets correlate with better health outcomes (108). A head-to-head comparison with the Mediterranean diet showed that plant-based diets increased faecal SCFAs and improved metabolic parameters (109). The benefits of plant-based diets depend on appropriate dietary planning to ensure nutritional adequacy (106).

Two caveats apply when generalising these findings to older adults. First, most cohorts documenting favourable microbial and cardiometabolic profiles in vegetarian or vegan populations are dominated by middle-aged rather than geriatric participants, and few include frail or sarcopenic elderly. Second, the metabolic advantages of plant-based patterns must be balanced against the increased protein requirements of older adults, in whom sarcopenia is a competing priority (see Section 5.4). Plant-based patterns are not, in themselves, contraindicated in older adults, but should incorporate deliberately higher-density protein sources (legumes, soy protein isolates, nuts, and where acceptable, dairy or eggs) with an even per-meal distribution reaching ≥0.4 g/kg per meal, in line with recent geriatric consensus statements (110). Vitamin B12, vitamin D, iron, zinc, calcium, and long-chain omega-3 fatty acid status also warrant periodic biochemical monitoring in older adults following a strict vegan pattern.

Intermittent fasting and metabolic flexibility. Metabolic flexibility refers to the body’s capacity to switch between glucose oxidation and fatty acid oxidation according to energy supply and demand. This capacity declines with ageing and metabolic syndrome and is an important feature of metabolic impairment in older adults. Intermittent fasting (IF) promotes metabolic switching through cycles of feeding and fasting, and has been shown to improve multiple cardiometabolic markers (111). Periodic fasting is thought to help delay age-related diseases (112).

Part of the benefit of intermittent fasting is mediated by the gut microbiota. Fasting remodels microbial circadian rhythms and increases microbial diversity, often accompanied by an increase in beneficial taxa such as *Akkermansia muciniphila* (113, 114). In patients with metabolic syndrome, intermittent fasting improves cardiometabolic risk factors and alters microbiota composition (115). Animal and human studies indicate that intermittent fasting modulates the microbiota and improves obesity and host energy metabolism, thereby enhancing metabolic flexibility (116). An umbrella review of randomised trials supports a role for intermittent fasting in improving obesity-related health outcomes (117).

Safety considerations for older adults. Extended fasting windows can precipitate hypoglycaemia in older adults on insulin, sulphonylureas, or SGLT2 inhibitors, and prolonged energy restriction may accelerate loss of lean mass in individuals already at risk of sarcopenia or unintentional weight loss (110). Time-restricted eating protocols with feeding windows of ≥10 h and protein-adequate meals are preferable in this population, and intermittent fasting should generally be avoided in patients with body mass index <22 kg/m^2^, recent unintentional weight loss >5%, or advanced frailty. Fluid intake, hypotension risk, and adjustment of anti-diabetic medication schedules also warrant explicit consideration before initiation.

Taken together, dietary patterns are a controllable lever on the gut–inflammageing–cardiometabolic axis. The Western diet accelerates inflammageing through dysbiosis, barrier leakage and endotoxaemia. The Mediterranean, plant-based and intermittent fasting patterns work in the opposite direction: they enrich SCFA-producing bacteria, reinforce barrier function, reduce pro-inflammatory and pro-thrombotic metabolites, and improve metabolic flexibility in older adults. The strength of the underlying evidence, however, is uneven, and the practical implementation of these patterns in frail older adults requires careful attention to protein adequacy, safety, and individual comorbidity profiles as discussed above and in Section 5.4. These observations support dietary intervention as a defensible approach to slow cardiometabolic ageing, although long-term effects and individualised regimens in older populations require further investigation (see Figure 5; Table 2).

**Figure 5:**
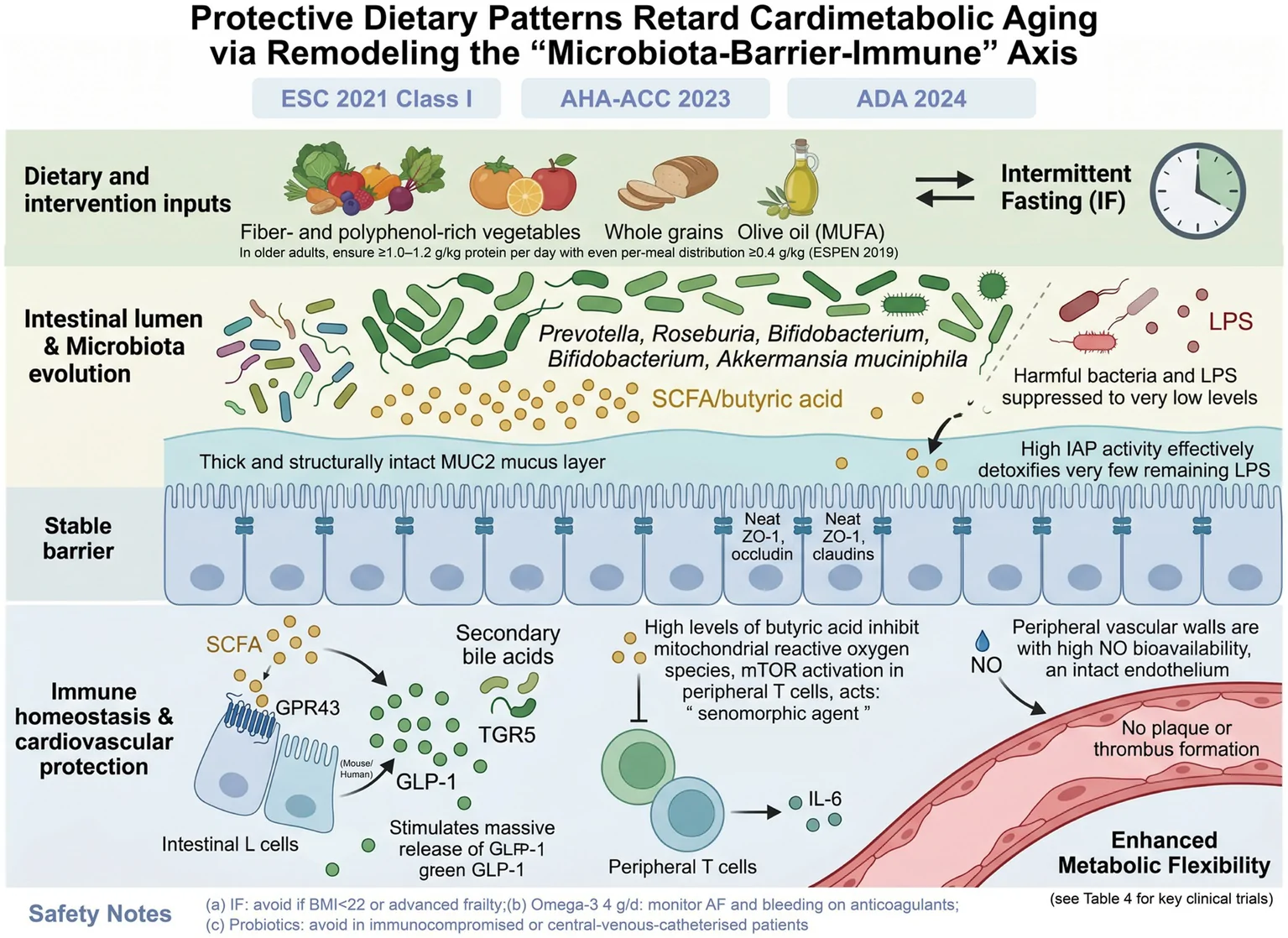
Protective dietary patterns retard cardiometabolic ageing via remodelling of the “microbiota–barrier–immune” axis. Schematic showing how protective dietary strategies, including Mediterranean-style intake rich in fibre, polyphenols, whole grains and olive oil, together with appropriately supervised intermittent fasting (IF), may remodel the gut ecosystem and protect the ageing cardiovascular system. In the intestinal lumen, these patterns enrich beneficial taxa, including *Prevotella*, *Roseburia*, *Bifidobacterium* and *Akkermansia muciniphila*, increase SCFA and butyrate production, and suppress LPS-producing pathobionts. Higher intestinal alkaline phosphatase (IAP) activity detoxifies residual LPS, while a structurally intact MUC2 mucus layer and preserved tight junctions maintain barrier integrity. SCFAs activate GPR43 on intestinal L cells, and secondary bile acids activate TGR5, promoting GLP-1 release and metabolic flexibility. Butyrate suppresses mitochondrial ROS and mTOR activation in peripheral T cells, reduces IL-6 output and acts as a senomorphic agent. Increased NO bioavailability supports endothelial integrity and limits plaque and thrombus formation. In older adults, these interventions require safety safeguards, including adequate protein intake, avoidance of IF in people with low body mass index (BMI) or advanced frailty, monitoring for atrial fibrillation (AF) and bleeding during high-dose omega-3 supplementation, and caution with probiotics in immunocompromised or central-venous-catheterised patients.

**Table 2:** Dietary patterns and their effects on the gut–inflammageing–cardiometabolic axis in older adults.

Dietary pattern	Compositional features	Effect on the gut–inflammageing–cardiometabolic axis	Level of evidence	Refs
Western diet	High saturated fat, refined sugar, high salt, red and processed meat; low dietary fibre	↓ SCFA producers; ↑ endotoxin-producing Gram-negative bacteria (e.g., *E. coli*); mucus and tight-junction disruption; postprandial LPS ↑ → TLR4/NF-κB activation; ↑ TMAO; accelerated inflammageing	Cohort + mechanistic RCTs	(52, 69, 75, 90, 97, 98)
Mediterranean diet	High fibre, extra-virgin olive oil (MUFAs), polyphenols, nuts, legumes, fish; moderate wine	Enriches *Faecalibacterium prausnitzii* and *Roseburia*; ↑ SCFAs; ↓ CRP and IL-17; improved frailty and cognitive indices; partly mediated via microbiota (particularly in low *Prevotella copri* phenotypes)	1-year RCT (NU-AGE) + large cohorts	(16, 31, 99–105)
Green-Mediterranean diet	Standard Mediterranean pattern plus Mankai duckweed and green tea; reduced red-meat intake	Distinct microbial remodelling associated with reductions in visceral adiposity and cardiometabolic risk factors independent of weight loss	18-month RCT (DIRECT-PLUS)	(17)
Plant-based (vegetarian / vegan)	Fibre- and polyphenol-rich; low or absent animal protein	↑ Microbial diversity and SCFA producers; ↑ faecal SCFAs; ↓ inflammatory markers; improved lipid and glycaemic profiles	Systematic review + large multi-cohort (>20,000)	(106–109)
Intermittent fasting (IF)	Time-restricted or alternate-day energy restriction; no fixed macronutrient prescription	Restores microbial circadian rhythm; enriches *Akkermansia muciniphila*; enhances metabolic flexibility (glucose↔fatty-acid oxidation); improves cardiometabolic risk in metabolic syndrome	RCTs + umbrella review	(111–117)

SCFA, short-chain fatty acid; CRP, C-reactive protein; LPS, lipopolysaccharide; TMAO, trimethylamine-N-oxide; MUFA, monounsaturated fatty acid; RCT, randomised controlled trial.

## 5 Key dietary components and molecular mechanisms

### 5.1 Dietary fibre and short-chain fatty acids: barrier maintenance and GPCR-mediated anti-inflammation

The receptor specificity of individual short-chain fatty acids adds a further layer of precision to their signalling. Acetate and propionate preferentially engage GPR43 (FFAR2), which is highly expressed on enteroendocrine L cells, adipocytes and neutrophils; propionate is a more potent GPR41 (FFAR3) agonist on sympathetic ganglia and portal vein afferents; and butyrate is the principal ligand of GPR109A (HCAR2), which also recognises niacin and β-hydroxybutyrate (118). This differential engagement helps explain why the three SCFAs, despite being structurally similar and metabolically related, produce partially non-overlapping physiological effects: propionate acting through GPR43 on L cells couples fibre intake to postprandial PYY and GLP-1 release, butyrate acting through GPR109A on colonic dendritic cells and macrophages promotes IL-10-producing regulatory T cells, and acetate reaching systemic circulation modulates hypothalamic AMPK and appetite regulation (27, 119). Interventions that shift the acetate:propionate:butyrate ratio therefore reshape the physiological read-out even when total SCFA production is preserved (118).

The HDAC-inhibitory action of butyrate has been reconceptualised in the framework of immunometabolism. In colonocytes, butyrate is the preferred mitochondrial substrate and its β-oxidation drives oxidative phosphorylation, stabilising a low-oxygen state at the epithelial apex that maintains commensal anaerobic communities (120, 121). In peripheral immune cells, class I HDAC inhibition by butyrate not only alters chromatin acetylation but also modulates the balance between glycolysis and oxidative phosphorylation, biasing differentiation of regulatory T cells and memory T cells toward oxidative phosphorylation while attenuating the glycolytic programme characteristic of effector Th17 and pro-inflammatory macrophages (122, 123). This immunometabolic action provides a mechanistic account of why SCFA-mediated tolerance is durable rather than transient (119).

The connection between SCFAs and mitochondrial quality control is increasingly evident. Butyrate promotes the expression of PGC-1α, the master regulator of mitochondrial biogenesis, in colonic and skeletal muscle tissue, supports the removal of damaged mitochondria through PINK1/Parkin-dependent mitophagy, and reduces mitochondrial reactive oxygen species (mtROS) generation in senescent cells (124, 125). Because mitochondrial dysfunction is itself a canonical hallmark of ageing, and because senescent cells with dysfunctional mitochondria are major contributors to the SASP, the mitochondrial actions of SCFAs suggest a mechanism by which dietary fibre exerts effects that extend well beyond the intestine (19, 67). The recent characterisation of butyrate as a ‘senomorphic’ agent that suppresses SASP output in senescent T cells rests substantially on these mitochondrial actions (124).

Fermentation of resistant starch, β-glucans and arabinoxylans yields different SCFA ratios and engages different microbial guilds, and the composition of the fibre matrix therefore shapes downstream signalling (126, 127). Resistant starch type 2 favours butyrate producers such as *Ruminococcus bromii* and *Faecalibacterium prausnitzii*; β-glucans from oats and barley are metabolised primarily by *Roseburia* and *Eubacterium rectale*, yielding a mixed acetate–butyrate profile; and inulin-type fructans support *Bifidobacterium* expansion and acetate production, with cross-feeding to butyrate producers via lactate and acetate exchange (126). Recognising fibre as a heterogeneous class rather than a single nutrient is essential for translating the mechanistic evidence into dietary recommendations for older adults, in whom the capacity to ferment complex substrates may itself be reduced (127).

Critical appraisal and clinical translation. Human intervention trials with isolated fibre supplements or SCFA precursors in older adults have shown small-to-moderate improvements in surrogate markers (fasting glucose, LDL-C, hs-CRP), but hard cardiovascular endpoints have not been demonstrated for any single fibre class in this population. A Cochrane meta-analysis of 55 RCTs of inulin-type fructans reported modest reductions in LDL-C, triglycerides and body weight with low-to-very-low certainty of evidence (128). Current dietary guidelines (2021 ESC, 2024 ADA, WHO 2023 dietary fibre recommendations) endorse an overall daily fibre intake of ≥25 g/day for women and ≥30 g/day for men, without preferential recommendation of specific fibre classes. In older adults, gradual titration of fibre intake is prudent to avoid gastrointestinal intolerance, and clinicians should recognise that age-related reduction in fermentation capacity may blunt biochemical responses to fibre supplementation.

### 5.2 Polyphenols: microbiota-derived metabolites as the anti-ageing effector

Polyphenols engage multiple host receptors and transcription factors beyond the direct anti-oxidant actions traditionally emphasised. The aryl hydrocarbon receptor (AhR) is a promiscuous sensor that recognises a broad spectrum of polyphenol-derived microbial metabolites, including urolithins, equol and enterolactone (129). AhR activation induces cytochrome P450 detoxification enzymes, sustains intraepithelial lymphocyte populations, and reinforces IL-22 production by type 3 innate lymphoid cells, which in turn supports epithelial regeneration and antimicrobial peptide expression (130). In older adults, in whom AhR ligand availability is reduced as a consequence of altered microbial metabolism, restoration of AhR tone through polyphenol-rich diets represents a plausible mechanistic route to reinforcing barrier and immune function (129).

The nuclear factor erythroid 2-related factor 2 (Nrf2) pathway is a further convergent target of polyphenol action. Under basal conditions Nrf2 is sequestered by KEAP1 and undergoes proteasomal degradation; polyphenol-derived electrophilic metabolites modify reactive cysteines in KEAP1, permit Nrf2 nuclear translocation, and induce a battery of antioxidant response element (ARE)-driven genes including HO-1, NQO1 and glutamate-cysteine ligase (131). This response mitigates the oxidative burden of ageing tissues and dampens the redox amplification of NF-κB and NLRP3 signalling. The connection between Nrf2 activation and mitochondrial biogenesis via PGC-1α cross-talk provides an additional link to the immunometabolic themes developed in Section 5.1 (125, 131).

The sirtuin family, particularly SIRT1 and SIRT3, is engaged by several polyphenol metabolites, most prominently the resveratrol-derived dihydroresveratrol and lunularin (132). Sirtuins deacetylate key transcription factors including p53, FOXO3a and PGC-1α, and their activation coordinates mitochondrial biogenesis, autophagy induction, and suppression of pro-inflammatory NF-κB acetylation (133). Because sirtuin activity declines with age in an NAD^+^-dependent manner, and because caloric restriction—the archetypal anti-ageing intervention—relies substantially on sirtuin activation, polyphenol-mediated sirtuin engagement offers a mechanistic bridge between dietary strategies and the core biology of ageing (133).

The urolithin story illustrates the depth of inter-individual variability in polyphenol biology. Only a subset of adults, so-called ‘urolithin producers’, harbour the microbial consortia—including certain *Gordonibacter* and *Ellagibacter* species—required to convert ellagitannins to urolithin A (134). Non-producers derive substantially less benefit from ellagitannin-rich foods such as pomegranates and walnuts, but respond in preliminary trials to direct urolithin A supplementation (124). This dichotomy is a paradigmatic example of the general problem of polyphenol response heterogeneity and provides a concrete case in which microbiota-informed precision nutrition has clear translational relevance (135).

The isoflavone-to-equol pathway offers a parallel example. Only about 30–50% of adults in Western populations, but a higher proportion in some East Asian populations, harbour the microbial equol-producing capacity encoded chiefly by strains of *Adlercreutzia equolifaciens* and *Slackia isoflavoniconvertens* (136). Equol has substantially higher oestrogen receptor β affinity and antioxidant potency than its parent daidzein, and equol producer status is associated with better cardiometabolic and bone health outcomes in ageing populations, particularly post-menopausal women (136). Together with the urolithin data, this observation reinforces the general principle that the phenotypic benefit of a given dietary polyphenol is contingent on the microbial genotype of the consumer (135).

Critical appraisal and clinical translation. Although mechanistic evidence for AhR-, Nrf2- and sirtuin-mediated actions of polyphenol metabolites is compelling, clinical trials of polyphenol-rich foods or extracts in older adults have produced inconsistent results on cardiometabolic endpoints. Effect sizes are typically small, heterogeneity across polyphenol classes (flavonoids, stilbenes, lignans, ellagitannins) is high, and the microbial capacity to generate the active metabolites—urolithins, equol, enterolactone—varies substantially between individuals. No polyphenol supplement is currently endorsed by cardiovascular or diabetes guidelines. Whole-food polyphenol intake (via fruits, vegetables, olive oil, tea, coffee), as recommended within Mediterranean or plant-based patterns, remains the most defensible approach; isolated high-dose polyphenol supplements should be considered investigational.

### 5.3 Omega-3 polyunsaturated fatty acids: membrane remodelling and resolution of inflammation

The receptor pharmacology of specialised pro-resolving mediators has been substantially clarified. Resolvin E1 signals through ChemR23 (also termed CMKLR1) and BLT1; resolvin D1 acts through ALX/FPR2 and GPR32; maresin 1 engages LGR6; and protectin D1 activates GPR37 (137, 138). These receptors are expressed on neutrophils, macrophages, and dendritic cells and, upon engagement, promote a switch from a pro-inflammatory to a pro-resolving phenotype characterised by efferocytosis of apoptotic neutrophils, non-phlogistic macrophage clearance, and secretion of anti-inflammatory mediators such as TGF-β and IL-10. Impaired expression or function of these receptors in ageing tissues represents an under-recognised mechanism of failed resolution and may contribute to the persistence of low-grade inflammation despite adequate omega-3 substrate availability (139).

The generation of SPMs is enzymatically coordinated and involves sequential lipoxygenase and cyclooxygenase steps. The initiating step for the resolvin E series is aspirin-acetylated COX-2, which redirects EPA metabolism toward 18R-HEPE; subsequent 5-LOX action yields RvE1 and RvE2. The D-series resolvins, protectins and maresins derive from DHA through 15-LOX and 12-LOX activity (138). The stereospecificity of these transformations makes SPM biosynthesis vulnerable to disturbances in enzyme expression and cofactor availability, both of which are altered with age, and low-dose aspirin has been proposed to potentiate omega-3-mediated resolution by biasing COX-2 toward the aspirin-acetylated form, a mechanism that is under active clinical investigation in cardiovascular prevention (139).

The clinical evidence for omega-3 fatty acids in older adults has evolved considerably. REDUCE-IT (*n* = 8,179 statin-treated adults, median follow-up 4.9 years) reported a 25% reduction in major adverse cardiovascular events with high-dose icosapent ethyl (4 g/day) in secondary prevention (140), whereas trials of mixed EPA/DHA formulations, including STRENGTH (*n* = 13,078, 42-month median follow-up) and VITAL (*n* = 25,871, ≥50 years, 5.3-year follow-up), produced null or modest results (141, 142). The heterogeneity is now attributed in part to differences in formulation, dose, and the omega-6 fatty acid content of the comparator, but also to individual variability in SPM biosynthetic capacity, and circulating SPM concentrations rather than parent fatty acid levels correlate more closely with clinical benefit in exploratory analyses (143). This observation reinforces the principle that intermediate metabolites, not substrates alone, define the biological response to nutritional interventions.

Safety considerations for older adults. Although omega-3 supplementation is generally well tolerated, REDUCE-IT and pooled post-hoc analyses of large omega-3 trials have highlighted a small but reproducible signal of increased incident atrial fibrillation and clinically relevant bleeding at 4 g/day of prescription-grade EPA or EPA/DHA. This risk is amplified in older adults on dual antiplatelet therapy, direct oral anticoagulants, or vitamin K antagonists, in whom dose titration together with monitoring of atrial rhythm and bleeding events is warranted. Whole-food omega-3 intake (fatty fish 2–3 servings per week) provides a safer route in most older adults and is the intake pattern recommended by the 2021 ESC and 2024 ADA guidelines (13, 15).

### 5.4 Protein intake and metabolism: requirements in older adults and the dual role of intestinal amino acid metabolites

The mechanistic distinction between anabolic signalling and inflammatory signalling by amino acids is central to understanding protein biology in older adults. Leucine and other branched-chain amino acids (BCAAs) engage mTORC1 through the Rag GTPase–Ragulator complex on the lysosomal surface, activating S6K1 and 4E-BP1 to drive protein synthesis (144). In skeletal muscle this action counters sarcopenia, but sustained mTORC1 activation in insulin-sensitive tissues promotes IRS-1 serine phosphorylation, uncouples insulin signalling, and contributes to hepatic insulin resistance (145). Microbially generated imidazole propionate further reinforces mTORC1 tone through p38γ MAPK-dependent phosphorylation of IRS1, providing a specific molecular route by which dysbiosis may exacerbate amino acid-driven insulin resistance (146). Balancing anabolic benefit against metabolic risk therefore requires attention not only to protein amount but to its temporal distribution and to the accompanying microbial metabolic context (145).

The tryptophan metabolic network extends beyond the indole route. Host indoleamine 2,3-dioxygenase (IDO1) diverts tryptophan into the kynurenine pathway, generating kynurenine, kynurenic acid, and quinolinic acid; these metabolites also engage AhR but with distinct biological consequences, including modulation of NAD^+^ biosynthesis through the *de novo* pathway (147). The kynurenine/tryptophan ratio rises with age and correlates with cognitive decline, muscle atrophy and vascular stiffness (148). Because gut bacteria compete with host IDO1 for tryptophan substrate, microbial indole production and host kynurenine generation are reciprocally related, and dietary tryptophan availability influences both (149, 150). This tripartite tryptophan–indole–kynurenine axis is emerging as a central hub linking diet, microbiota, immune ageing, and neurocognitive function (147).

The sulphur amino acid axis provides a further example of dual-action metabolism. Cysteine and methionine feed both the transsulphuration pathway, which sustains glutathione biosynthesis and cellular redox homeostasis, and the microbial generation of hydrogen sulphide (H₂S) (151). At physiological concentrations H₂S functions as a gasotransmitter that supports mitochondrial bioenergetics, vasodilation, and epithelial mucus production; at supra-physiological concentrations, generated for example by expansion of sulphate-reducing *Desulfovibrio* spp. on a high-animal-protein diet, H₂S impairs colonocyte β-oxidation and compromises barrier integrity (151). The concentration-dependent switch from beneficial to detrimental effects captures a broader principle in nutritional biology: many microbial metabolites are ‘hormetic’ molecules whose signalling reverses across a concentration threshold that is itself shifted by ageing (90).

Plant protein sources such as legumes, nuts and soy alter the substrate profile presented to the colonic microbiota. Compared with animal protein, plant protein delivers a higher proportion of indigestible protein fractions to the colon accompanied by fibre and polyphenols, favouring saccharolytic over proteolytic fermentation and reducing the generation of TMA precursors (90). Substitution studies in older adults have reported that replacing red and processed meat with plant protein lowers circulating TMAO, improves lipid profiles, and modestly reduces inflammatory markers (152). These findings do not argue for eliminating animal protein in the elderly, in whom sarcopenia risk is a competing priority (see also Section 4.3), but do support a shift in the balance of protein sources as one of the more actionable dietary levers in this population (145). The ESPEN 2019 clinical guideline on nutrition in geriatrics recommends a daily protein intake of 1.0–1.2 g/kg for healthy older adults and 1.2–1.5 g/kg for those with acute or chronic illness, with a per-meal target of ≥0.4 g/kg to maximally stimulate muscle protein synthesis (110). Achieving this target within a predominantly plant-based pattern is feasible but requires deliberate meal planning around high-density plant proteins (soy, legumes, nuts, whole grains) or supplementary dairy and eggs where acceptable.

As summarised in Table 3, the four classes of components discussed in this Section share a common feature: their activity is largely mediated by microbial metabolites, with converging targets at GPCRs, AhR, NF-κB and components of mitochondrial quality control. Whether these mechanisms translate into stable clinical effects in older populations will require further interventional evidence in adequately powered, geriatric-representative trials with hard clinical endpoints. The corresponding intervention strategies, safety considerations in frail older adults, and current guideline landscape are examined systematically in Section 6.

**Table 3:** Key dietary bioactive components: Microbial intermediates, molecular targets and effects on inflammageing.

Component	Representative food sources	Microbial/Metabolic intermediates	Molecular targets and effects on inflammageing	Refs
Dietary fibre (resistant starch, β-glucans, inulin, arabinoxylans)	Whole grains, legumes, oats, barley, chicory root, cooked-and-cooled starches	Acetate, propionate, butyrate; substrate-specific enrichment of *Ruminococcus bromii*, *F. prausnitzii*, *Roseburia*, *Eubacterium rectale*, *Bifidobacterium*	GPR43/GPR41/GPR109A engagement → L-cell GLP-1 and PYY release; regulatory T-cell induction (butyrate); PGC-1α ↑ and mitophagy support; class I HDAC inhibition; senomorphic suppression of SASP	(27, 28, 118–123, 125–127)
Polyphenols	Berries, pomegranate, walnuts, tea, cocoa, extra-virgin olive oil, soy, red wine	Urolithin A, equol, enterolactone; dihydroresveratrol, lunularin; producer status depends on *Gordonibacter*, *Ellagibacter*, *Adlercreutzia equolifaciens*, *Slackia isoflavoniconvertens*	AhR activation → IL-22, epithelial regeneration, antimicrobial peptides; Nrf2/KEAP1 → HO-1, NQO1, ARE-gene induction; SIRT1/SIRT3 activation → deacetylation of p53, FOXO3a, PGC-1α; suppression of NF-κB and NLRP3	(100, 124, 129–136)
Omega-3 PUFAs (EPA, DHA)	Oily fish (salmon, mackerel, sardines), fish oil, algal oil, flaxseed (α-linolenic acid precursor)	Resolvin E1/E2 (from EPA via aspirin-acetylated COX-2 and 5-LOX); resolvin D-series, protectins, maresins (from DHA via 15-LOX and 12-LOX)	ChemR23/BLT1 (RvE1), ALX/FPR2 and GPR32 (RvD1), LGR6 (MaR1), GPR37 (PD1) engagement → efferocytosis, non-phlogistic macrophage clearance, TGF-β and IL-10 secretion; circulating SPM concentrations predict cardiovascular benefit	(137–143)
Dietary protein and amino acids	Plant sources (legumes, nuts, soy) vs. animal sources (red / processed meat, dairy, eggs, fish)	Leucine and BCAAs; imidazole propionate; indoles vs. kynurenine (host IDO1); H^2^S from *Desulfovibrio* spp.; TMA precursors from carnitine/choline	mTORC1/S6K1/4E-BP1 activation → anabolic muscle support but IRS-1 serine phosphorylation and hepatic insulin resistance; imidazole propionate reinforces mTORC1 via p38γ MAPK; AhR modulation via tryptophan–indole–kynurenine axis; hormetic H₂S signalling in colonocytes	(90, 110, 144–152)

PUFA, polyunsaturated fatty acid; EPA, eicosapentaenoic acid; DHA, docosahexaenoic acid; SPM, specialised pro-resolving mediator; RvE/RvD, resolvin E/D-series; MaR1, maresin 1; PD1, protectin D1; BCAA, branched-chain amino acid; AhR, aryl hydrocarbon receptor; Nrf2, nuclear factor erythroid 2-related factor 2; SIRT, sirtuin; HDAC, histone deacetylase; SASP, senescence-associated secretory phenotype; IRS-1, insulin receptor substrate 1; GLP-1, glucagon-like peptide-1; PYY, peptide YY.

## 6 Interventions and translational perspectives

The preceding Sections described the mechanistic link between the gut microbiota, inflammageing and cardiometabolic disorders. This Section turns to intervention and translation, examining how these mechanisms can be converted into actionable prevention and treatment strategies (80). Slowing inflammageing and cardiometabolic ageing is a central goal in the context of population ageing (21). Five sub-topics are examined in turn: precision nutrition based on individual microbiota profiles (Section 6.1), biotherapeutics including probiotics, prebiotics, and postbiotics (Section 6.2), small-molecule drugs that target specific metabolites (Section 6.3), safety considerations relevant to frail older adults (Section 6.4), and the translation of these approaches into current clinical guidelines and practice (Section 6.5).

### 6.1 Precision nutrition: tailored diets informed by individual microbiota profiles

The premise of precision nutrition is that individuals respond differently to the same foods. Large cohort studies show that postprandial glycaemic and lipid responses to identical meals vary substantially between individuals, and the gut microbiota is one of the determinants of this variability (153). Microbiota composition itself can also predict several cardiometabolic markers to some extent (104). Multi-omics analyses suggest that the relationship among diet, microbiota and cardiometabolic health carries a strong individual-specific component (103).

This premise has been translated into operational interventions. Machine-learning models built on individual microbiota profiles and clinical features can predict postprandial glycaemic responses and inform tailored diets. Several randomised controlled trials have provided initial evidence, though sample sizes and follow-up durations remain modest. A randomised pilot trial (*n* = 20 newly diagnosed patients with type 2 diabetes, 6-month intervention) reported that diets personalised by predicted glycaemic responses improved glycaemic control and cardiometabolic markers relative to a Mediterranean comparator (154). A randomised controlled trial in 225 adults with prediabetes (6-month intervention) compared a personalised postprandial-glucose-targeting (PPT) diet with a Mediterranean diet and reported a greater reduction in glycaemic variability and HbA1c with the PPT arm (155). A larger randomised controlled trial in 347 adults from the general population (18-week intervention) showed that a personalised nutrition programme integrating glycaemic, triglyceride, microbiota, and health-history information produced modest but statistically significant improvements in postprandial triglycerides, insulin, and self-reported gut health versus the US dietary guidelines arm (156). Overall these trials are pilot- to modest-scale investigations with intermediate biomarker endpoints; effect sizes are small-to-moderate, hard cardiovascular endpoints have not yet been examined, and applicability to frail older adults has not been formally tested. Adding microbiota data to predictive models can enhance the accuracy of individual glycaemic response prediction (157).

Precision nutrition nevertheless faces well-recognised translational challenges that currently limit routine clinical implementation. First, there is no consensus on stool sampling, storage, DNA extraction, sequencing platform (16S versus shotgun), taxonomic classifier, or bioinformatics pipeline, and inter-laboratory reproducibility remains low; the same sample can yield materially different taxonomic profiles across platforms. Second, individual microbiome profiles show substantial temporal instability, particularly in older adults, so a single time-point measurement may not reliably characterise habitual composition. Third, large prospective validation studies with hard cardiometabolic endpoints, adequate representation of older and non-Western populations, and pre-registered analysis plans are still scarce, and commercial claims often outpace the underlying evidence (158). Fourth, per-participant costs of shotgun metagenomics and comprehensive metabolomics remain prohibitive for widespread screening. Fifth, translational infrastructure—clinical-grade laboratory certification, actionable-report standards, and dietitian and prescriber training—has not been established. Application in older adults should therefore proceed cautiously, remain research-embedded, and be guided by high-quality evidence. Key clinical trials referenced in this Section are summarised in Table 4.

**Table 4:** Summary of major clinical trials and meta-analyses cited in this review.

Study/Ref	Design	Population (n, age, setting)	Intervention	Duration	Primary outcome
DIRECT-PLUS (17)	Single-centre RCT	294 adults with abdominal obesity, mean 51 y (Israel)	Green-Mediterranean vs. Mediterranean vs. healthy dietary guidelines	18 months	Microbiota remodelling; cardiometabolic risk markers
REDUCE-IT (140)	Multicentre RCT	8,179 statin-treated adults, ≥45 y	Icosapent ethyl 4 g/d vs. placebo	Median 4.9 y	MACE ↓25%; ↑ AF, ↑ bleeding
STRENGTH (141)	Multicentre RCT	13,078 statin-treated adults	Omega-3 CA 4 g/d vs. corn oil	Median 42 months	No MACE benefit
VITAL (142)	Placebo-controlled RCT	25,871 adults ≥50 y	Omega-3 1 g/d ± vit D vs. placebo	Median 5.3 y	No primary MACE benefit
NU-AGE (31)	Multicentre RCT	Older adults 65–79 y (5 European countries)	Mediterranean diet vs. habitual diet	12 months	Microbiota shifts; frailty & inflammation markers ↓
Rein et al. (154)	Pilot RCT	20 newly diagnosed T2DM patients	Personalised diet (algorithm) vs. Mediterranean	6 months	Postprandial glucose; HbA1c
Ben-Yacov et al. (155)	RCT	225 adults with prediabetes	PPT-personalised vs. Mediterranean	6 months	HbA1c; glucose variability
ZOE PREDICT / Bermingham et al. (156)	RCT	347 general-population adults	Personalised nutrition programme vs. US dietary guidelines	18 weeks	Postprandial triglycerides; insulin; gut health
Kocsis et al. (160)	Meta-analysis (32 RCTs)	Adults with T2DM	Probiotics (heterogeneous strains) vs. placebo	4 wk. – 6 mo	TC, TG, CRP, HbA1c, FPG ↓
Li et al. (161)	Meta-analysis (30 RCTs)	1,827 patients with T2DM	Probiotics vs. placebo	Variable	FPG, HbA1c, HOMA-IR ↓
Talukdar et al. (128)	Meta-analysis (55 RCTs)	2,518 participants	Inulin-type fructans vs. placebo/other	Variable	LDL, TG, BW ↓; low certainty

CV, cardiovascular; MACE, major adverse cardiovascular events; AF, atrial fibrillation; EVOO, extra-virgin olive oil; PPT, postprandial-glucose-targeting; FPG, fasting plasma glucose; TC, total cholesterol; TG, triglyceride; BW, body weight.

### 6.2 Probiotics, prebiotics and postbiotics: adjuncts to pharmacotherapy

The three categories of biotherapeutics need to be defined. Probiotics are live microorganisms that confer health benefits to the host when administered in adequate amounts. Prebiotics are substrates that promote the growth of beneficial bacteria. Postbiotics are defined as “preparations of inanimate microorganisms and/or their components that confer a health benefit on the host” (159).

Clinical evidence has accumulated for probiotics as adjuncts to therapy in type 2 diabetes. A meta-analysis of 32 randomised controlled trials reported that probiotics lowered total cholesterol, triglycerides, C-reactive protein, glycated haemoglobin, fasting glucose and insulin, and reduced both systolic and diastolic blood pressure (160). A separate meta-analysis of 30 RCTs (1,827 patients with T2DM) reported that probiotic supplementation improved glycaemic control, lowering fasting glucose, glycated haemoglobin and HOMA-IR (161). These improvements in cardiometabolic and inflammatory markers support a possible role for probiotics as adjuncts to glucose- and lipid-lowering therapy.

Prebiotics act mainly through their fermentation products. Inulin-type fructans (including inulin, fructo-oligosaccharides and galacto-oligosaccharides) are fermented by the microbiota to produce short-chain fatty acids. A meta-analysis of 55 RCTs (2,518 participants) reported that inulin-type fructan supplementation may reduce LDL cholesterol, triglycerides and body weight, with little to no measurable effect on other cardiovascular risk factors; the certainty of evidence was rated low to very low (128). The metabolic benefits are likely mediated by short-chain fatty acids (27).

Postbiotics are an emerging direction, with short-chain fatty acids as a representative class. Butyrate and related postbiotics strengthen the gut barrier, suppress inflammation, and induce regulatory T cells (122). These actions provide a mechanistic basis for clinical translation, and clinical research on butyrate is ongoing (123).

Taken together, probiotics, prebiotics and postbiotics may serve as adjuncts to conventional pharmacotherapy for diabetes and cardiovascular disease in older adults. The clinical evidence, however, has important limitations that must be flagged. First, probiotic effects are strongly strain-specific; pooled meta-analyses treating heterogeneous strains (Lactobacillus, Bifidobacterium, Streptococcus, Saccharomyces and multi-strain formulations) as a single class obscure clinically meaningful strain-level differences. Second, trials differ substantially in participant characteristics (metabolically healthy, prediabetic, T2DM with variable HbA1c, on-treatment vs. treatment-naïve), dose (10^7^–10^11^ CFU/day), delivery matrix (capsule, yoghurt, fermented milk), and duration (4 weeks to 6 months), so aggregate estimates carry considerable heterogeneity. Third, effects on hard endpoints—cardiovascular events, incident T2DM, all-cause mortality—have not been demonstrated for any probiotic in adequately powered trials. Fourth, current major cardiovascular and diabetes guidelines (2021 ESC Cardiovascular Disease Prevention, 2023 AHA/ACC Chronic Coronary Disease, 2024 ADA Standards of Care) do not include a formal recommendation on probiotic, prebiotic, or postbiotic use for cardiometabolic prevention or management (13–15). Larger, longer-term, strain-specific randomised trials with pre-registered protocols and geriatric representation are needed before firm clinical recommendations can be made (128, 160, 161).

### 6.3 Targeting gut metabolites: small molecules that block TMAO production or mimic SCFA action

Compared with modulating the microbiota as a whole, targeting specific metabolites offers greater precision: block the production of harmful metabolites, or mimic the action of beneficial ones.

Trimethylamine N-oxide (TMAO) is the main target of this strategy. TMAO is regarded as an independent risk marker for cardiovascular disease (90). An umbrella review with an updated meta-analysis reported a dose–response relationship between TMAO and several adverse health outcomes (162), and an association with stroke risk (163). Small-molecule interventions have focused on the upstream steps of TMAO production, largely in preclinical models. The choline structural analogue 3,3-dimethyl-1-butanol (DMB) non-lethally inhibits microbial trimethylamine (TMA) lyases, reduces TMA and TMAO formation, and attenuates atherosclerosis in ApoE-deficient mice (164). Mechanism-based inhibitors such as iodomethylcholine target choline TMA-lyase with greater specificity in murine and cell-based systems (164). Inhibition of host flavin-containing monooxygenase 3 (FMO3) also lowers TMAO, but the approach is limited by hepatic inflammation and a “fish-odour syndrome” phenotype, which constrain its value as a drug target (164, 165). Human interventional data on TMAO-lowering small molecules remain limited, and none of these compounds is currently approved for clinical use. Dietary strategies can also reduce circulating TMAO; for example, a vegan diet lowers plasma TMAO (166).

Mimicking SCFA action represents another route. The idea is to supplement butyrate and related SCFAs, or to activate their receptors (such as GPR43/FFAR2 and GPR109A), in order to restore beneficial signalling. SCFAs strengthen the gut barrier, suppress NF-κB, and induce regulatory T cells (27, 122). Clinical translation of butyrate is being explored in early-phase human studies (123). Direct small-molecule mimicry of SCFA signalling in humans still rests on limited evidence.

The three routes are conceptually complementary. Precision nutrition advises an individual on what to eat. Biotherapeutics decide which microbes or microbial products to supply. Small-molecule drugs adjust specific metabolites. Most of the metabolite-targeting evidence remains derived from animal models and early human studies, and the safety and effectiveness of TMAO-lowering compounds and SCFA mimetics in older adults will require testing in adequately powered clinical trials before any translational recommendation can be made.

### 6.4 Safety considerations and contraindications in frail older adults

The interventions discussed above are generally well tolerated in metabolically healthy middle-aged adults, but their risk–benefit balance in frail older adults warrants specific consideration before translation into geriatric practice (110).

Intermittent fasting. Extended fasting windows can precipitate hypoglycaemia in older adults on insulin, sulphonylureas, or SGLT2 inhibitors, and prolonged energy restriction may accelerate loss of lean mass in individuals already at risk of sarcopenia or unintentional weight loss. Time-restricted eating protocols with feeding windows of ≥10 h and protein-adequate meals are preferable in this population, and intermittent fasting should generally be avoided in patients with body mass index <22 kg/m^2^, recent unintentional weight loss >5%, or advanced frailty. Fluid intake, hypotension risk, and adjustment of glucose-lowering medication schedules should be reviewed before initiation (see also Section 4.3).

High-dose omega-3 / EPA supplementation. REDUCE-IT and STRENGTH, together with post-hoc analyses of VITAL, have highlighted a small but reproducible signal of increased incident atrial fibrillation and clinically relevant bleeding at 4 g/day of prescription-grade EPA or EPA/DHA (140–142). This risk is amplified in older adults on dual antiplatelet therapy, direct oral anticoagulants, or vitamin K antagonists, in whom dose titration together with monitoring of atrial rhythm and bleeding events is warranted. Whole-food omega-3 intake (fatty fish 2–3 servings per week) provides a safer route in most older adults and is the intake pattern recommended by the 2021 ESC and 2024 ADA guidelines (13, 15).

Probiotics. Although the overall safety record of commercial probiotic strains is excellent, rare cases of bacteraemia and fungaemia have been documented in immunocompromised, critically ill, or central-venous-catheterised patients, particularly with Saccharomyces boulardii and Lactobacillus species (167). Probiotics should therefore be avoided in older adults during acute hospitalisation with central lines, in those undergoing chemotherapy or receiving high-dose immunosuppression, and in patients with recent gastrointestinal surgery.

Plant-based dietary patterns. As discussed in Sections 4.3 and 5.4, vegan patterns adopted without dietary planning may lead to protein, vitamin B12, vitamin D, calcium, iron, zinc, and long-chain omega-3 insufficiency, all of which are already at heightened risk in older adults. Supervision by a registered dietitian and periodic biochemical monitoring are recommended, with explicit attention to a per-meal protein target of ≥0.4 g/kg to preserve muscle mass (110).

TMAO-lowering small molecules. Compounds such as 3,3-dimethyl-1-butanol (DMB) and iodomethylcholine remain investigational; long-term safety data in humans, and especially in older adults with polypharmacy, renal impairment, or hepatic dysfunction, are not yet available. Their use should currently be restricted to clinical trials.

In summary, the interventions discussed in Sections 4–6 should be introduced into geriatric clinical practice with explicit attention to frailty status, comorbidities, concomitant medications, and nutritional adequacy. A tiered, individualised approach—starting with dietary pattern optimisation, adding biotherapeutics selectively, and reserving metabolite-targeted small molecules for research settings—is currently the most defensible translational stance.

### 6.5 Translation into current clinical guidelines and practice

Existing cardiovascular and diabetes practice guidelines have begun to incorporate elements of the axis reviewed here, though they remain conservative regarding microbiota-targeted interventions.

The 2021 ESC Guidelines on Cardiovascular Disease Prevention give a Class I recommendation to a Mediterranean-style dietary pattern for both primary and secondary prevention (13); the 2023 AHA/ACC/ACCP/ASPC/NLA/PCNA Guideline for the Management of Patients with Chronic Coronary Disease (14) and the 2024 ADA Standards of Care in Diabetes (15) similarly endorse Mediterranean, DASH, and plant-forward eating patterns as first-line lifestyle interventions.

By contrast, current guidelines do not yet recommend routine microbiota profiling, personalised nutrition on the basis of gut microbiota data, or the use of specific probiotic or prebiotic products for cardiovascular or diabetes prevention. Neither the ESC nor the ADA endorses TMAO as a routine clinical biomarker, and small molecules targeting TMAO biosynthesis remain investigational (13, 15).

The ESPEN 2019 guideline on clinical nutrition and hydration in geriatrics recommends a protein intake of 1.0–1.2 g/kg per day for healthy older adults and 1.2–1.5 g/kg per day for those with acute or chronic illness, with even per-meal distribution and, where possible, high-quality protein sources (110)—a recommendation that must be integrated with any plant-based dietary counselling in this population.

Practically, the mechanistic evidence reviewed here supports the following clinical actions in older adults: (i) prioritising Mediterranean or Green-Mediterranean dietary patterns with age-appropriate protein density; (ii) considering probiotics as adjuncts rather than replacements for guideline-directed pharmacotherapy in type 2 diabetes and hyperlipidaemia; (iii) restricting personalised microbiome-guided nutrition and TMAO-lowering compounds to research or expert-centre settings until validation data mature; and (iv) integrating a formal frailty assessment before recommending intermittent fasting or vegan patterns (see Section 6.4). This tiered approach aligns mechanistic ambition with what current guidelines currently permit.

## 7 Conclusion and future directions

### 7.1 Summary

This review has followed one organising thread: the gut microbiota, the intestinal barrier, the immune system, and cardiometabolic homeostasis form a modifiable feedback loop, and diet is the most direct and actionable entry point into that loop (74, 80). The earlier Sections traced this loop step by step. Dysbiosis can disrupt the barrier and produce metabolic endotoxaemia, which sustains a low-grade systemic inflammation and feeds into vascular injury through insulin resistance and metabolites such as TMAO (80, 85). The same processes overlap heavily with ageing-related inflammageing (19, 21).

Dietary components act on the host largely through microbial metabolites and represent one of the few reversible nodes on this loop. Dietary fibre maintains the barrier and induces regulatory T cells through short-chain fatty acids (SCFAs) (27). The activity of polyphenols depends to a considerable extent on their microbial metabolites (100). Omega-3 polyunsaturated fatty acids contribute to the resolution of inflammation through membrane remodelling and specialised pro-resolving mediators (137). The NU-AGE study in older adults across five European countries reported that 1 year of Mediterranean diet enriched SCFA-producing bacteria, with improvements in frailty indices and inflammatory markers (31). Diet acts on this loop in two directions, shaping microbial composition and metabolite output upstream and influencing the barrier, immunity, and vascular function downstream. This dual action, together with the clinical actions identified in Section 6.5 and the safety framework outlined in Section 6.4, positions dietary intervention as a defensible—if still incompletely validated—entry point for slowing cardiometabolic ageing.

### 7.2 Research gaps

The mechanistic evidence is fairly coherent. Translation into geriatric clinical practice still leaves several gaps, which extend beyond those already highlighted in Section 6.

Long-term follow-up is scarce. Most randomised controlled trials in the diet–microbiota–cardiometabolic field span 8 to 24 weeks (17). Studies of 1 year or more that use cardiovascular events or frailty progression as hard endpoints are still few; NU-AGE remains one of the more representative examples (31). Without longer follow-up, whether the observed improvements in microbial and metabolic markers translate into survival and functional benefit cannot yet be settled.

Ethnic and genetic differences are under-recognised. Large cohorts have mainly been drawn from European and North American populations (92). Microbial composition and dietary baselines differ systematically in Chinese, South Asian, African, and Latin American populations. Extrapolation from Western data should therefore be made with caution. The transferability of microbiota-informed precision nutrition across ethnic groups awaits validation in local cohorts (158).

Heterogeneity across ageing stages is overlooked. Healthy older adults, pre-frail individuals and frail individuals differ in microbial composition, barrier function and anabolic response (19, 21). The benefit and risk of the same intervention may not be equivalent across these stages. Most trials stratify participants by chronological age and rarely by frailty phenotype, which limits a finer assessment of intervention benefit. Explicit frailty stratification—for example, using the Fried phenotype or the Clinical Frailty Scale—should be a routine requirement of future trials.

Evidence on causal direction is limited. Cross-sectional and short-term interventional studies dominate. Tools capable of establishing causal direction in large populations, such as Mendelian randomisation and microbiota transplantation trials, are still limited in number and largely restricted to specialist centres (92, 103). Where causal claims are made, this review has explicitly qualified them—particularly where the underlying evidence is derived from a single rodent model or an observational cohort (see, for example, Sections 2.1 and 3.3.2).

Geriatric safety data are scarce. Most trials of intermittent fasting, high-dose omega-3, and probiotics either exclude the oldest-old or under-report frailty-specific adverse events (hypoglycaemia, sarcopenia, bleeding, rare bacteraemia in immunocompromised individuals). Prospective registries or trial extensions designed to capture such events in geriatric cohorts are needed before broader clinical adoption (167).

### 7.3 Future outlook

A reasonable medium-to-long-term direction is to incorporate gut microbiota assessment into routine screening and nutritional intervention for cardiometabolic disease in older adults. This vision should be developed at three levels while remaining aligned with current guideline evidence (13–15, 110).

Screening. In cardiometabolic risk assessment, a panel of microbial and metabolite indicators could complement conventional lipid, glucose, and inflammatory measures. Such a panel might include faecal 16S rRNA diversity indices, the relative abundance of butyrate-producing bacteria, and circulating TMAO and LPS concentrations (85, 90). Clinical thresholds and reproducibility for these indicators, however, still need to be established in prospective cohorts before they can be recommended for routine use.

Intervention. Beyond routine nutritional counselling, personalised dietary regimens based on integrated microbial and clinical features can be introduced step by step. Trials such as DIRECT-PLUS and ZOE PREDICT provide pilot evidence in support of this approach (17, 156) (see Table 4). Implementation in geriatric practice should be paired with the safety framework outlined in Section 6.4 and the guideline-based hierarchy summarised in Section 6.5.

Implementation. Three parallel efforts will determine whether this goal can be realised. The first is long-term randomised trials with hard endpoints in geriatric-representative populations. The second is reference cohorts that cover different ethnic groups and ageing stages. The third is methodological standardisation, including shared operating procedures for sample handling, sequencing, and bioinformatics analysis (103, 158). A fourth priority—dedicated safety surveillance in frail older adults—should be added to this list, as noted in Section 7.2.

Bringing dietary intervention, biotherapeutics, and small-molecule tools targeting microbial metabolites into a single clinical decision framework, testing this framework in long-term trials, and coupling it with an explicit safety and guideline-integration layer (Sections 6.4 and 6.5), is the next step in turning the existing mechanistic evidence into broadly applicable practice.

## References

[1] Qu C Liao S Zhang J Cao H Zhang H Zhang N . Burden of cardiovascular disease among elderly: based on the global burden of disease study 2019. Eur Heart J Qual Care Clin Outcomes. (2024) 10:143–53. doi: 10.1093/ehjqcco/qcad033, 37296238 PMC10904724

[2] Chong B Jayabaskaran J Jauhari SM Chan SP Goh R Kueh MTW . Global burden of cardiovascular diseases: projections from 2025 to 2050. Eur J Prev Cardiol. (2025) 32:1001–15. doi: 10.1093/eurjpc/zwae281, 39270739

[3] Karpuzoglu E Holladay SD Gogal RM Jr. Inflammaging: triggers, molecular mechanisms, immunological consequences, sex differences, and cutaneous manifestations. Front Immunol. (2025) 16:1704203. doi: 10.3389/fimmu.2025.1704203, 41425557 PMC12714610

[4] Abdellatif M Rainer PP Sedej S Kroemer G. Hallmarks of cardiovascular ageing. Nat Rev Cardiol. (2023) 20:754–77. doi: 10.1038/s41569-023-00881-3, 37193857

[5] Aranda JF Ramírez CM Mittelbrunn M. Inflammageing, a targetable pathway for preventing cardiovascular diseases. Cardiovasc Res. (2025) 121:1537–50. doi: 10.1093/cvr/cvae240, 39530590 PMC12391671

[6] Ghosh TS Shanahan F O'Toole PW. The gut microbiome as a modulator of healthy ageing. Nat Rev Gastroenterol Hepatol. (2022) 19:565–84. doi: 10.1038/s41575-022-00605-x, 35468952 PMC9035980

[7] Caetano-Silva ME Shrestha A Duff AF Kontic D Brewster PC Kasperek MC . Aging amplifies a gut microbiota immunogenic signature linked to heightened inflammation. Aging Cell. (2024) 23:e14190. doi: 10.1111/acel.14190, 38725282 PMC11320341

[8] Chae YR Lee YR Kim YS Park HY. Diet-induced gut dysbiosis and leaky gut syndrome. J Microbiol Biotechnol. (2024) 34:747–56. doi: 10.4014/jmb.2312.12031, 38321650 PMC11091682

[9] Escalante J Artaiz O Diwakarla S McQuade RM. Leaky gut in systemic inflammation: exploring the link between gastrointestinal disorders and age-related diseases. Geroscience. (2025) 47:1–22. doi: 10.1007/s11357-024-01451-2, 39638978 PMC11872833

[10] Canyelles M Borràs C Rotllan N Tondo M Escolà-Gil JC Blanco-Vaca F. Gut microbiota-derived TMAO: a causal factor promoting atherosclerotic cardiovascular disease? Int J Mol Sci. (2023) 24:1940. doi: 10.3390/ijms24031940, 36768264 PMC9916030

[11] Giangregorio F Mosconi E Debellis MG Provini S Esposito C Garolfi M . A systematic review of metabolic syndrome: key correlated pathologies and non-invasive diagnostic approaches. J Clin Med. (2024) 13:5880. doi: 10.3390/jcm13195880, 39407941 PMC11478146

[12] Holmannova D Borsky P Andrys C Kremlacek J Fiala Z Parova H . The influence of metabolic syndrome on potential aging biomarkers in participants with metabolic syndrome compared to healthy controls. Biomedicine. (2024) 12:242. doi: 10.3390/biomedicines12010242, 38275413 PMC10813522

[13] Visseren FLJ Mach F Smulders YM Carballo D. 2021 ESC guidelines on cardiovascular disease prevention in clinical practice. Eur Heart J. (2021) 42:3227–337. doi: 10.1093/eurheartj/ehab484, 34458905

[14] Virani SS Newby LK Arnold SV Bittner V Brewer LC Demeter SH . AHA/ACC/ACCP/ASPC/NLA/PCNA guideline for the management of patients with chronic coronary disease: a report of the American Heart Association/American College of Cardiology Joint Committee on clinical practice guidelines. J Am Coll Cardiol. (2023) 82:833–955. doi: 10.1016/j.jacc.2023.04.003 37480922

[15] Committee, A.D.A.P.P. Diagnosis and classification of diabetes: standards of care in diabetes-2024. Diabetes Care. (2024) 47:S20–42. doi: 10.2337/dc24-S002 PMC10725812 38078589

[16] Tessier AJ Wang F Korat AA Eliassen AH Chavarro J Grodstein F . Optimal dietary patterns for healthy aging. Nat Med. (2025) 31:1644–52. doi: 10.1038/s41591-025-03570-5, 40128348 PMC12092270

[17] Rinott E Meir AY Tsaban G Zelicha H Kaplan A Knights D . The effects of the green-Mediterranean diet on cardiometabolic health are linked to gut microbiome modifications: a randomized controlled trial. Genome Med. (2022) 14:29. doi: 10.1186/s13073-022-01015-z, 35264213 PMC8908597

[18] Cano R Bermúdez V Galban N Garrido B Santeliz R Gotera MP . Dietary polyphenols and gut microbiota cross-talk: molecular and therapeutic perspectives for cardiometabolic disease: a narrative review. Int J Mol Sci. (2024) 25:9118. doi: 10.3390/ijms25169118, 39201807 PMC11354808

[19] López-Otín C Blasco MA Partridge L Serrano M Kroemer G. Hallmarks of aging: An expanding universe. Cell. (2023) 186:243–78. doi: 10.1016/j.cell.2022.11.001, 36599349

[20] DeJong EN Surette MG Bowdish DME. The gut microbiota and unhealthy aging: disentangling cause from consequence. Cell Host Microbe. (2020) 28:180–9. doi: 10.1016/j.chom.2020.07.013, 32791111

[21] Conway J. Ageing of the gut microbiome: potential influences on immune senescence and inflammageing. Ageing Res Rev. (2021) 68:101323. doi: 10.1016/j.arr.2021.101323 33771720

[22] Bosco N Noti M. The aging gut microbiome and its impact on host immunity. Genes Immun. (2021) 22:289–303. doi: 10.1038/s41435-021-00126-8, 33875817 PMC8054695

[23] Wilmanski T Diener C Rappaport N Patwardhan S Wiedrick J Lapidus J . Gut microbiome pattern reflects healthy ageing and predicts survival in humans. Nat Metab. (2021) 3:274–86. doi: 10.1038/s42255-021-00348-0, 33619379 PMC8169080

[24] Sato Y Atarashi K Plichta DR Arai Y. Novel bile acid biosynthetic pathways are enriched in the microbiome of centenarians. Nature. (2021) 599:458–64. doi: 10.1038/s41586-021-03832-5, 34325466

[25] Leite G Pimentel M Barlow GM Chang C Hosseini A Wang J . Age and the aging process significantly alter the small bowel microbiome. Cell Rep. (2021) 36:109765. doi: 10.1016/j.celrep.2021.109765, 34592155

[26] Le Cosquer G Vergnolle N Motta JP. Gut microb-aging and its relevance to frailty aging. Microbes Infect. (2024) 26:105309. doi: 10.1016/j.micinf.2024.105309, 38316374

[27] Du Y He C An Y Huang Y Zhang H Fu W . The role of short chain fatty acids in inflammation and body health. Int J Mol Sci. (2024) 25:7379. doi: 10.3390/ijms25137379, 39000498 PMC11242198

[28] Rees NP Conway J Dugan B Amir SS Parker A Carding SR . Defining microbiota-derived metabolite butyrate as a Senomorphic: therapeutic potential in the age-related T cell senescence. Aging Cell. (2025) 24:e70257. doi: 10.1111/acel.70257, 41201238 PMC12686592

[29] Parker A Romano S Ansorge R Aboelnour A Le Gall G Savva GM . Fecal microbiota transfer between young and aged mice reverses hallmarks of the aging gut, eye, and brain. Microbiome. (2022) 10:68. doi: 10.1186/s40168-022-01243-w, 35501923 PMC9063061

[30] You X Dadwal UC Lenburg ME Kacena MA Charles JF. Murine gut microbiome meta-analysis reveals alterations in carbohydrate metabolism in response to aging. mSystems. (2022) 7:e0124821. doi: 10.1128/msystems.01248-21, 35400171 PMC9040766

[31] Ghosh TS Rampelli S Jeffery IB Santoro A Neto M Capri M . Mediterranean diet intervention alters the gut microbiome in older people reducing frailty and improving health status: the NU-AGE 1-year dietary intervention across five European countries. Gut. (2020) 69:1218–28. doi: 10.1136/gutjnl-2019-319654, 32066625 PMC7306987

[32] Schmidt V Enav H Spector TD Youngblut ND Ley RE. Strain-level analysis of *Bifidobacterium* spp. from gut microbiomes of adults with differing lactase persistence genotypes. mSystems. (2020) 5:e00911-20. doi: 10.1128/mSystems.00911-20, 32994293 PMC7527142

[33] Frazier K Chang EB. Intersection of the gut microbiome and circadian rhythms in metabolism. Trends Endocrinol Metab. (2020) 31:25–36. doi: 10.1016/j.tem.2019.08.013, 31677970 PMC7308175

[34] Teichman EM O'Riordan KJ Gahan CGM Dinan TG Cryan JF. When rhythms meet the blues: circadian interactions with the microbiota-gut-brain Axis. Cell Metab. (2020) 31:448–71. doi: 10.1016/j.cmet.2020.02.008, 32130879

[35] Xia J Guo W Hu M Jin X Zhang S Liu B . Resynchronized rhythmic oscillations of gut microbiota drive time-restricted feeding induced nonalcoholic steatohepatitis alleviation. Gut Microbes. (2023) 15:2221450. doi: 10.1080/19490976.2023.2221450, 37309179 PMC10266122

[36] Salazar AM Aparicio R Clark RI Rera M Walker DW. Intestinal barrier dysfunction: an evolutionarily conserved hallmark of aging. Dis Model Mech. (2023) 16:dmm049969. doi: 10.1242/dmm.049969, 37144684 PMC10184675

[37] Sang X Wang Q Ning Y Wang H Zhang R Li Y . Age-related mucus barrier dysfunction in mice is related to the changes in Muc 2 mucin in the colon. Nutrients. (2023) 15:1830. doi: 10.3390/nu15081830, 37111049 PMC10145456

[38] Neurath MF Artis D Becker C. The intestinal barrier: a pivotal role in health, inflammation, and cancer. Lancet Gastroenterol Hepatol. (2025) 10:573–92. doi: 10.1016/s2468-1253(24)00390-x, 40086468

[39] Ahmad R Kumar B Thapa I Talmon GA Salomon J Ramer-Tait AE . Loss of claudin-3 expression increases colitis risk by promoting gut dysbiosis. Gut Microbes. (2023) 15:2282789. doi: 10.1080/19490976.2023.2282789, 38010872 PMC10730149

[40] Ghosh SS Wang J Yannie PJ Ghosh S. Intestinal barrier dysfunction, LPS translocation, and disease development. J Endocr Soc. (2020) 4:bvz039. doi: 10.1210/jendso/bvz039, 32099951 PMC7033038

[41] Gong CX Ma C Irge DD Li SM Chen SM Zhou SX . *Gastrodia elata* and parishin ameliorate aging induced 'leaky gut' in mice: correlation with gut microbiota. Biom J. (2023) 46:100547. doi: 10.1016/j.bj.2022.07.001, 35811058 PMC10345228

[42] He WQ Wang J Sheng JY Zha JM Graham WV Turner JR. Contributions of myosin light chain kinase to regulation of epithelial paracellular permeability and mucosal homeostasis. Int J Mol Sci. (2020) 21:993. doi: 10.3390/ijms21030993, 32028590 PMC7037368

[43] Brandt A Baumann A Hernández-Arriaga A Jung F Nier A Staltner R . Impairments of intestinal arginine and NO metabolisms trigger aging-associated intestinal barrier dysfunction and 'inflammaging'. Redox Biol. (2022) 58:102528. doi: 10.1016/j.redox.2022.102528, 36356464 PMC9649383

[44] Nalapareddy K Nattamai KJ Kumar RS Karns R Wikenheiser-Brokamp KA Sampson LL . Canonical Wnt Signaling ameliorates aging of intestinal stem cells. Cell Rep. (2017) 18:2608–21. doi: 10.1016/j.celrep.2017.02.056, 28297666 PMC5987258

[45] Nalapareddy K Zheng Y Geiger H. Aging of intestinal stem cells. Stem Cell Reports. (2022) 17:734–40. doi: 10.1016/j.stemcr.2022.02.003, 35276089 PMC9023768

[46] Sovran B Hugenholtz F Elderman M Van Beek AA Graversen K Huijskes M . Age-associated impairment of the mucus barrier function is associated with profound changes in microbiota and immunity. Sci Rep. (2019) 9:1437. doi: 10.1038/s41598-018-35228-3, 30723224 PMC6363726

[47] Knoop KA Gustafsson JK McDonald KG Kulkarni DH Coughlin PE McCrate S . Microbial antigen encounter during a preweaning interval is critical for tolerance to gut bacteria. Sci Immunol. (2017) 2:eaao1314. doi: 10.1126/sciimmunol.aao1314, 29246946 PMC5759965

[48] Untersmayr E Brandt A Koidl L Bergheim I. The intestinal barrier dysfunction as driving factor of inflammaging. Nutrients. (2022) 14:949. doi: 10.3390/nu14050949, 35267924 PMC8912763

[49] Saccon TD Nagpal R Yadav H Cavalcante MB Nunes ADC Schneider A . Senolytic combination of Dasatinib and quercetin alleviates intestinal senescence and inflammation and modulates the gut microbiome in aged mice. J Gerontol A Biol Sci Med Sci. (2021) 76:1895–905. doi: 10.1093/gerona/glab002, 33406219 PMC8514064

[50] Islam MT Tuday E Allen S Kim J Trott DW Holland WL . Senolytic drugs, dasatinib and quercetin, attenuate adipose tissue inflammation, and ameliorate metabolic function in old age. Aging Cell. (2023) 22:e13767. doi: 10.1111/acel.13767, 36637079 PMC9924942

[51] Camilleri M. Leaky gut: mechanisms, measurement and clinical implications in humans. Gut. (2019) 68:1516–26. doi: 10.1136/gutjnl-2019-318427, 31076401 PMC6790068

[52] Mohammad S Thiemermann C. Role of metabolic endotoxemia in systemic inflammation and potential interventions. Front Immunol. (2020) 11:594150. doi: 10.3389/fimmu.2020.594150, 33505393 PMC7829348

[53] Leu SY Tsang YL Ho LC Yang CC Shao AN Chang CY . NLRP3 inflammasome activation, metabolic danger signals, and protein binding partners. J Endocrinol. (2023) 257:e220184. doi: 10.1530/joe-22-0184, 36799299

[54] Sebastian-Valverde M Pasinetti GM. The NLRP3 inflammasome as a critical actor in the inflammaging process. Cells. (2020) 9:1552. doi: 10.3390/cells9061552, 32604771 PMC7348816

[55] Zhang Y Zhang S Li B Luo Y Gong Y Jin X . Gut microbiota dysbiosis promotes age-related atrial fibrillation by lipopolysaccharide and glucose-induced activation of NLRP3-inflammasome. Cardiovasc Res. (2022) 118:785–97. doi: 10.1093/cvr/cvab114, 33757127

[56] Tylutka A Walas Ł Zembron-Lacny A. Level of IL-6, TNF, and IL-1β and age-related diseases: a systematic review and meta-analysis. Front Immunol. (2024) 15:1330386. doi: 10.3389/fimmu.2024.1330386, 38495887 PMC10943692

[57] Prokopidis K Giannos P Kirwan R Ispoglou T Galli F Witard OC . Impact of probiotics on muscle mass, muscle strength and lean mass: a systematic review and meta-analysis of randomized controlled trials. J Cachexia Sarcopenia Muscle. (2023) 14:30–44. doi: 10.1002/jcsm.13132, 36414567 PMC9891957

[58] Sharma BR Kanneganti TD. NLRP3 inflammasome in cancer and metabolic diseases. Nat Immunol. (2021) 22:550–9. doi: 10.1038/s41590-021-00886-5, 33707781 PMC8132572

[59] Yi YS. Roles of the Caspase-11 non-canonical inflammasome in rheumatic diseases. Int J Mol Sci. (2024) 25. doi: 10.3390/ijms25042091, 38396768 PMC10888639

[60] Hopfner KP Hornung V. Molecular mechanisms and cellular functions of cGAS-STING signalling. Nat Rev Mol Cell Biol. (2020) 21:501–21. doi: 10.1038/s41580-020-0244-x 32424334

[61] Glück S Guey B Gulen MF Wolter K Kang TW Schmacke NA . Innate immune sensing of cytosolic chromatin fragments through cGAS promotes senescence. Nat Cell Biol. (2017) 19:1061–70. doi: 10.1038/ncb3586, 28759028 PMC5826565

[62] Zhong W Rao Z Xu J Sun Y Hu H Wang P . Defective mitophagy in aged macrophages promotes mitochondrial DNA cytosolic leakage to activate STING signaling during liver sterile inflammation. Aging Cell. (2022) 21:e13622. doi: 10.1111/acel.13622, 35599014 PMC9197407

[63] Jaiswal S Natarajan P Silver AJ Gibson CJ Bick AG Shvartz E . Clonal Hematopoiesis and risk of atherosclerotic cardiovascular disease. N Engl J Med. (2017) 377:111–21. doi: 10.1056/NEJMoa1701719, 28636844 PMC6717509

[64] Thevaranjan N Puchta A Schulz C Naidoo A Szamosi JC Verschoor CP . Age-associated microbial dysbiosis promotes intestinal permeability, systemic inflammation, and macrophage dysfunction. Cell Host Microbe. (2017) 21:455–466.e4. doi: 10.1016/j.chom.2017.03.002, 28407483 PMC5392495

[65] Ezenabor EH Adeyemi AA Adeyemi OS. Gut microbiota and metabolic syndrome: relationships and opportunities for new therapeutic strategies. Scientifica (Cairo). (2024) 2024:4222083. doi: 10.1155/2024/4222083, 39041052 PMC11262881

[66] Sasidharan Pillai S Gagnon CA Foster C Ashraf AP. Exploring the gut microbiota: key insights into its role in obesity, metabolic syndrome, and type 2 diabetes. J Clin Endocrinol Metab. (2024) 109:2709–19. doi: 10.1210/clinem/dgae499, 39040013 PMC11479700

[67] Baechle JJ Chen N Makhijani P Winer S Furman D Winer DA. Chronic inflammation and the hallmarks of aging. Mol Metab. (2023) 74:101755. doi: 10.1016/j.molmet.2023.101755, 37329949 PMC10359950

[68] Cani PD Van Hul M. Gut microbiota in overweight and obesity: crosstalk with adipose tissue. Nat Rev Gastroenterol Hepatol. (2024) 21:164–83. doi: 10.1038/s41575-023-00867-z, 38066102

[69] Tilg H Zmora N Adolph TE Elinav E. The intestinal microbiota fuelling metabolic inflammation. Nat Rev Immunol. (2020) 20:40–54. doi: 10.1038/s41577-019-0198-4, 31388093

[70] Magne F Gotteland M Gauthier L Zazueta A Pesoa S Navarrete P . The Firmicutes/Bacteroidetes ratio: a relevant marker of gut dysbiosis in obese patients? Nutrients. (2020) 12:1474. doi: 10.3390/nu12051474, 32438689 PMC7285218

[71] Cunningham AL Stephens JW Harris DA. A review on gut microbiota: a central factor in the pathophysiology of obesity. Lipids Health Dis. (2021) 20:65. doi: 10.1186/s12944-021-01491-z, 34233682 PMC8262044

[72] Liu BN Liu XT Liang ZH Wang JH. Gut microbiota in obesity. World J Gastroenterol. (2021) 27:3837–50. doi: 10.3748/wjg.v27.i25.3837, 34321848 PMC8291023

[73] Aron-Wisnewsky J Warmbrunn MV Nieuwdorp M Clément K. Metabolism and metabolic disorders and the microbiome: the intestinal microbiota associated with obesity, lipid metabolism, and metabolic health-pathophysiology and therapeutic strategies. Gastroenterology. (2021) 160:573–99. doi: 10.1053/j.gastro.2020.10.057, 33253685

[74] Fan Y Pedersen O. Gut microbiota in human metabolic health and disease. Nat Rev Microbiol. (2021) 19:55–71. doi: 10.1038/s41579-020-0433-9 32887946

[75] Agus A Clément K Sokol H. Gut microbiota-derived metabolites as central regulators in metabolic disorders. Gut. (2021) 70:1174–82. doi: 10.1136/gutjnl-2020-323071, 33272977 PMC8108286

[76] Wani K Rahman S Draz H. Editorial: dysbiosis, obesity, and inflammation: interrelated phenomena causes or effects of metabolic syndrome? Front Endocrinol (Lausanne). (2023) 14:1265314. doi: 10.3389/fendo.2023.1265314, 37916148 PMC10616948

[77] Gurung M Li Z You H Rodrigues R Jump DB Morgun A . Role of gut microbiota in type 2 diabetes pathophysiology. EBioMedicine. (2020) 51:102590. doi: 10.1016/j.ebiom.2019.11.051, 31901868 PMC6948163

[78] Scheithauer TPM Rampanelli E Nieuwdorp M Vallance BA Verchere CB van Raalte DH . Gut microbiota as a trigger for metabolic inflammation in obesity and type 2 diabetes. Front Immunol. (2020) 11:571731. doi: 10.3389/fimmu.2020.571731, 33178196 PMC7596417

[79] Koh A Bäckhed F. From association to causality: the role of the gut microbiota and its functional products on host metabolism. Mol Cell. (2020) 78:584–96. doi: 10.1016/j.molcel.2020.03.005, 32234490

[80] de Vos WM Tilg H Van Hul M Cani PD. Gut microbiome and health: mechanistic insights. Gut. (2022) 71:1020–32. doi: 10.1136/gutjnl-2021-326789, 35105664 PMC8995832

[81] Dulai AS Min M Sivamani RK. The gut microbiome's influence on incretins and impact on blood glucose control. Biomedicine. (2024) 12:2719. doi: 10.3390/biomedicines12122719, 39767626 PMC11727616

[82] Cani PD Depommier C Derrien M Everard A de Vos WM. *Akkermansia muciniphila*: paradigm for next-generation beneficial microorganisms. Nat Rev Gastroenterol Hepatol. (2022) 19:625–37. doi: 10.1038/s41575-022-00631-9, 35641786

[83] Yang G Wei J Liu P Zhang Q Tian Y Hou G . Role of the gut microbiota in type 2 diabetes and related diseases. Metabolism. (2021) 117:154712. doi: 10.1016/j.metabol.2021.154712, 33497712

[84] Régnier M Van Hul M Knauf C Cani PD. Gut microbiome, endocrine control of gut barrier function and metabolic diseases. J Endocrinol. (2021) 248:R67–r82. doi: 10.1530/joe-20-0473, 33295880

[85] Witkowski M Weeks TL Hazen SL. Gut microbiota and cardiovascular disease. Circ Res. (2020) 127:553–70. doi: 10.1161/circresaha.120.316242, 32762536 PMC7416843

[86] Vourakis M Mayer G Rousseau G. The role of gut microbiota on cholesterol metabolism in atherosclerosis. Int J Mol Sci. (2021) 22:8074. doi: 10.3390/ijms22158074, 34360839 PMC8347163

[87] Kazemian N Mahmoudi M Halperin F Wu JC Pakpour S. Gut microbiota and cardiovascular disease: opportunities and challenges. Microbiome. (2020) 8:36. doi: 10.1186/s40168-020-00821-0, 32169105 PMC7071638

[88] Masenga SK Kabwe LS Chakulya M Kirabo A. Mechanisms of oxidative stress in metabolic syndrome. Int J Mol Sci. (2023) 24:7898. doi: 10.3390/ijms24097898, 37175603 PMC10178199

[89] Verhaar BJH Prodan A Nieuwdorp M Muller M. Gut microbiota in hypertension and atherosclerosis: a review. Nutrients. (2020) 12:2982. doi: 10.3390/nu12102982, 33003455 PMC7601560

[90] Zhen J Zhou Z He M Han HX Lv EH Wen PB . The gut microbial metabolite trimethylamine N-oxide and cardiovascular diseases. Front Endocrinol (Lausanne). (2023) 14:1085041. doi: 10.3389/fendo.2023.1085041, 36824355 PMC9941174

[91] Nemet I Saha PP Gupta N Zhu W Romano KA Skye SM . A cardiovascular disease-linked gut microbial metabolite acts via adrenergic receptors. Cell. (2020) 180:862–877.e22. doi: 10.1016/j.cell.2020.02.016, 32142679 PMC7402401

[92] Chakaroun RM Olsson LM Bäckhed F. The potential of tailoring the gut microbiome to prevent and treat cardiometabolic disease. Nat Rev Cardiol. (2023) 20:217–35. doi: 10.1038/s41569-022-00771-0, 36241728

[93] Brunt VE Gioscia-Ryan RA Casso AG VanDongen NS Ziemba BP Sapinsley ZJ . Trimethylamine-N-oxide promotes age-related vascular oxidative stress and endothelial dysfunction in mice and healthy humans. Hypertension. (2020) 76:101–12. doi: 10.1161/hypertensionaha.120.14759, 32520619 PMC7295014

[94] Brunt VE Casso AG Gioscia-Ryan RA Sapinsley ZJ Ziemba BP Clayton ZS . Gut microbiome-derived metabolite trimethylamine N-oxide induces aortic stiffening and increases systolic blood pressure with aging in mice and humans. Hypertension. (2021) 78:499–511. doi: 10.1161/hypertensionaha.120.16895, 33966451 PMC8266738

[95] Abbasalizad Farhangi M Vajdi M Nikniaz L Nikniaz Z. The interaction between dietary inflammatory index and 6 P21 rs2010963 gene variants in metabolic syndrome. Eat Weight Disord. (2020) 25:1049–60. doi: 10.1007/s40519-019-00729-1, 31197703

[96] Trøseid M Andersen G Broch K Hov JR. The gut microbiome in coronary artery disease and heart failure: current knowledge and future directions. EBioMedicine. (2020) 52:102649. doi: 10.1016/j.ebiom.2020.102649, 32062353 PMC7016372

[97] Malesza IJ Malesza M Walkowiak J Mussin N Walkowiak D Aringazina R . High-fat, western-style diet, systemic inflammation, and gut microbiota: a narrative review. Cells. (2021) 10:3164. doi: 10.3390/cells10113164, 34831387 PMC8619527

[98] Elizabeth L Machado P Zinöcker M Baker P Lawrence M. Ultra-processed foods and health outcomes: a narrative review. Nutrients. (2020) 12:1955. doi: 10.3390/nu12071955, 32630022 PMC7399967

[99] Wastyk HC Fragiadakis GK Perelman D Dahan D Merrill BD Yu FB . Gut-microbiota-targeted diets modulate human immune status. Cell. (2021) 184:4137–4153.e14. doi: 10.1016/j.cell.2021.06.019, 34256014 PMC9020749

[100] Wang X Qi Y Zheng H. Dietary polyphenol, gut microbiota, and health benefits. Antioxidants (Basel). (2022) 11:1212. doi: 10.3390/antiox11061212, 35740109 PMC9220293

[101] Meslier V Laiola M Roager HM De Filippis F Roume H Quinquis B . Mediterranean diet intervention in overweight and obese subjects lowers plasma cholesterol and causes changes in the gut microbiome and metabolome independently of energy intake. Gut. (2020) 69:1258–68. doi: 10.1136/gutjnl-2019-320438, 32075887 PMC7306983

[102] Wang DD Nguyen LH Li Y Yan Y Ma W Rinott E . The gut microbiome modulates the protective association between a Mediterranean diet and cardiometabolic disease risk. Nat Med. (2021) 27:333–43. doi: 10.1038/s41591-020-01223-3, 33574608 PMC8186452

[103] Valles-Colomer M Menni C Berry SE Valdes AM Spector TD Segata N. Cardiometabolic health, diet and the gut microbiome: a meta-omics perspective. Nat Med. (2023) 29:551–61. doi: 10.1038/s41591-023-02260-4, 36932240 PMC11258867

[104] Asnicar F Berry SE Valdes AM Nguyen LH Piccinno G Drew DA . Microbiome connections with host metabolism and habitual diet from 1,098 deeply phenotyped individuals. Nat Med. (2021) 27:321–32. doi: 10.1038/s41591-020-01183-8, 33432175 PMC8353542

[105] Hernando-Redondo J Hernáez Á Sanllorente A Pintó X Estruch R Salas-Salvadó J . Mediterranean diet modulates gene expression of cholesterol efflux receptors in high-risk cardiovascular patients. Mol Nutr Food Res. (2025) 69:e70050. doi: 10.1002/mnfr.70050, 40344371 PMC12236127

[106] Sidhu SRK Kok CW Kunasegaran T Ramadas A. Effect of plant-based diets on gut microbiota: a systematic review of interventional studies. Nutrients. (2023) 15:1510. doi: 10.3390/nu15061510, 36986240 PMC10057430

[107] Miao Z Du W Xiao C Su C Gou W Shen L . Gut microbiota signatures of long-term and short-term plant-based dietary pattern and cardiometabolic health: a prospective cohort study. BMC Med. (2022) 20:204. doi: 10.1186/s12916-022-02402-4, 35701845 PMC9199182

[108] Fackelmann G Manghi P Carlino N Heidrich V Piccinno G Ricci L . Gut microbiome signatures of vegan, vegetarian and omnivore diets and associated health outcomes across 21,561 individuals. Nat Microbiol. (2025) 10:41–52. doi: 10.1038/s41564-024-01870-z, 39762435 PMC11726441

[109] Pagliai G Russo E Niccolai E Dinu M Di Pilato V Magrini A . Influence of a 3-month low-calorie Mediterranean diet compared to the vegetarian diet on human gut microbiota and SCFA: the CARDIVEG study. Eur J Nutr. (2020) 59:2011–24. doi: 10.1007/s00394-019-02050-0, 31292752

[110] Volkert D Beck AM Cederholm T Cruz-Jentoft A Goisser S Hooper L . ESPEN guideline on clinical nutrition and hydration in geriatrics. Clin Nutr. (2019) 38:10–47. doi: 10.1016/j.clnu.2018.05.024, 30005900

[111] Varady KA Cienfuegos S Ezpeleta M Gabel K. Cardiometabolic benefits of intermittent fasting. Annu Rev Nutr. (2021) 41:333–61. doi: 10.1146/annurev-nutr-052020-041327, 34633860

[112] Longo VD Di Tano M Mattson MP Guidi N. Intermittent and periodic fasting, longevity and disease. Nat Aging. (2021) 1:47–59. doi: 10.1038/s43587-020-00013-3, 35310455 PMC8932957

[113] Popa AD Niță O Gherasim A Enache AI Caba L Mihalache L . A scoping review of the relationship between intermittent fasting and the human gut microbiota: current knowledge and future directions. Nutrients. (2023) 15:2095. doi: 10.3390/nu15092095, 37432222 PMC10180719

[114] Pieczyńska-Zając JM Malinowska A Łagowska K Leciejewska N Bajerska J. The effects of time-restricted eating and Ramadan fasting on gut microbiota composition: a systematic review of human and animal studies. Nutr Rev. (2024) 82:777–93. doi: 10.1093/nutrit/nuad093, 37528052 PMC11082590

[115] Guo Y Luo S Ye Y Yin S Fan J Xia M. Intermittent fasting improves cardiometabolic risk factors and alters gut microbiota in metabolic syndrome patients. J Clin Endocrinol Metab. (2021) 106:64–79. doi: 10.1210/clinem/dgaa644, 33017844

[116] Hu X Xia K Dai M Han X Yuan P Liu J . Intermittent fasting modulates the intestinal microbiota and improves obesity and host energy metabolism. NPJ Biofilms Microbiomes. (2023) 9:19. doi: 10.1038/s41522-023-00386-4, 37029135 PMC10081985

[117] Patikorn C Roubal K Veettil SK Chandran V Pham T Lee YY . Intermittent fasting and obesity-related health outcomes: An umbrella review of Meta-analyses of randomized clinical trials. JAMA Netw Open. (2021) 4:e2139558. doi: 10.1001/jamanetworkopen.2021.39558, 34919135 PMC8683964

[118] Priyadarshini M Kotlo KU Dudeja PK Layden BT. Role of short chain fatty acid receptors in intestinal physiology and pathophysiology. Compr Physiol. (2018) 8:1091–115. doi: 10.1002/cphy.c170050, 29978895 PMC6058973

[119] Martin-Gallausiaux C Marinelli L Blottière HM Larraufie P Lapaque N. SCFA: mechanisms and functional importance in the gut. Proc Nutr Soc. (2021) 80:37–49. doi: 10.1017/s0029665120006916, 32238208

[120] Byndloss MX Olsan EE Rivera-Chávez F Tiffany CR Cevallos SA Lokken KL . Microbiota-activated PPAR-γ signaling inhibits dysbiotic Enterobacteriaceae expansion. Science. (2017) 357:570–5. doi: 10.1126/science.aam9949, 28798125 PMC5642957

[121] Kelly CJ Zheng L Campbell EL Saeedi B Scholz CC Bayless AJ . Crosstalk between microbiota-derived short-chain fatty acids and intestinal epithelial HIF augments tissue barrier function. Cell Host Microbe. (2015) 17:662–71. doi: 10.1016/j.chom.2015.03.005, 25865369 PMC4433427

[122] Siddiqui MT Cresci GAM. The immunomodulatory functions of butyrate. J Inflamm Res. (2021) 14:6025–41. doi: 10.2147/jir.S300989, 34819742 PMC8608412

[123] Hodgkinson K El Abbar F Dobranowski P Manoogian J Butcher J Figeys D . Butyrate's role in human health and the current progress towards its clinical application to treat gastrointestinal disease. Clin Nutr. (2023) 42:61–75. doi: 10.1016/j.clnu.2022.10.024, 36502573

[124] Denk D Singh A Kasler HG D'Amico D Rey J Alcober-Boquet L . Effect of the mitophagy inducer urolithin a on age-related immune decline: a randomized, placebo-controlled trial. Nat Aging. (2025) 5:2309–22. doi: 10.1038/s43587-025-00996-x PMC12618261 41174221

[125] Rius-Pérez S Torres-Cuevas I Millán I Ortega Á L Pérez S. PGC-1α, inflammation, and oxidative stress: an integrative view in metabolism. Oxidative Med Cell Longev. (2020) 2020:1452696. doi: 10.1155/2020/1452696 PMC7085407 32215168

[126] Baxter NT Schmidt AW Venkataraman A Kim KS Waldron C Schmidt TM. Dynamics of human gut microbiota and short-chain fatty acids in response to dietary interventions with three fermentable fibers. MBio. (2019) 10:e02566-18. doi: 10.1128/mBio.02566-18, 30696735 PMC6355990

[127] Deehan EC Yang C Perez-Muñoz ME Nguyen NK Cheng CC Triador L . Precision microbiome modulation with discrete dietary Fiber structures directs short-chain fatty acid production. Cell Host Microbe. (2020) 27:389–404.e6. doi: 10.1016/j.chom.2020.01.006, 32004499

[128] Talukdar JR Cooper M Lyutvyn L Zeraatkar D Ali R Berbrier R . The effects of inulin-type fructans on cardiovascular disease risk factors: systematic review and meta-analysis of randomized controlled trials. Am J Clin Nutr. (2024) 119:496–510. doi: 10.1016/j.ajcnut.2023.10.030, 38309832

[129] Rothhammer V Quintana FJ. The aryl hydrocarbon receptor: an environmental sensor integrating immune responses in health and disease. Nat Rev Immunol. (2019) 19:184–97. doi: 10.1038/s41577-019-0125-8, 30718831

[130] Zelante T Iannitti RG Cunha C De Luca A Giovannini G Pieraccini G . Tryptophan catabolites from microbiota engage aryl hydrocarbon receptor and balance mucosal reactivity via interleukin-22. Immunity. (2013) 39:372–85. doi: 10.1016/j.immuni.2013.08.003, 23973224

[131] Yu C Xiao JH. The Keap1-Nrf2 system: a mediator between oxidative stress and aging. Oxidative Med Cell Longev. (2021) 2021:6635460. doi: 10.1155/2021/6635460, 34012501 PMC8106771

[132] Li F Han Y Wu X Cao X Gao Z Sun Y . Gut microbiota-derived resveratrol metabolites, Dihydroresveratrol and Lunularin, significantly contribute to the biological activities of resveratrol. Front Nutr. (2022) 9:912591. doi: 10.3389/fnut.2022.912591, 35634412 PMC9131081

[133] Chung S Yao H Caito S Hwang JW Arunachalam G Rahman I. Regulation of SIRT1 in cellular functions: role of polyphenols. Arch Biochem Biophys. (2010) 501:79–90. doi: 10.1016/j.abb.2010.05.003, 20450879 PMC2930135

[134] García-Villalba R Giménez-Bastida JA Cortés-Martín A Ávila-Gálvez M Tomás-Barberán FA Selma MV . Urolithins: a comprehensive update on their metabolism, bioactivity, and associated gut microbiota. Mol Nutr Food Res. (2022) 66:e2101019. doi: 10.1002/mnfr.202101019, 35118817 PMC9787965

[135] Cortés-Martín A Selma MV Tomás-Barberán FA González-Sarrías A Espín JC. Where to look into the puzzle of polyphenols and health? The postbiotics and gut microbiota associated with human Metabotypes. Mol Nutr Food Res. (2020) 64:e1900952. doi: 10.1002/mnfr.201900952, 32196920

[136] Setchell KD Clerici C. Equol: history, chemistry, and formation. J Nutr. (2010) 140:1355s–62s. doi: 10.3945/jn.109.119776, 20519412 PMC2884333

[137] Ji RR. Specialized pro-resolving mediators as resolution pharmacology for the control of pain and itch. Annu Rev Pharmacol Toxicol. (2023) 63:273–93. doi: 10.1146/annurev-pharmtox-051921-084047, 36100219 PMC10290889

[138] Chiang N Serhan CN. Specialized pro-resolving mediator network: an update on production and actions. Essays Biochem. (2020) 64:443–62. doi: 10.1042/ebc20200018, 32885825 PMC7682745

[139] Bäck M Yurdagul A Jr Tabas I Öörni K Kovanen PT. Inflammation and its resolution in atherosclerosis: mediators and therapeutic opportunities. Nat Rev Cardiol. (2019) 16:389–406. doi: 10.1038/s41569-019-0169-2, 30846875 PMC6727648

[140] Bhatt DL Steg PG Miller M Brinton EA Jacobson TA Ketchum SB . Cardiovascular risk reduction with Icosapent ethyl for hypertriglyceridemia. N Engl J Med. (2019) 380:11–22. doi: 10.1056/NEJMoa1812792, 30415628

[141] Nicholls SJ Lincoff AM Garcia M Bash D Ballantyne CM Barter PJ . Effect of high-dose Omega-3 fatty acids vs corn oil on major adverse cardiovascular events in patients at high cardiovascular risk: the STRENGTH randomized clinical trial. JAMA. (2020) 324:2268–80. doi: 10.1001/jama.2020.22258, 33190147 PMC7667577

[142] Manson JE Cook NR Lee IM Christen W Bassuk SS Mora S . Marine n-3 fatty acids and prevention of cardiovascular disease and Cancer. N Engl J Med. (2019) 380:23–32. doi: 10.1056/NEJMoa1811403, 30415637 PMC6392053

[143] Barden AE Shinde S Burke V Puddey IB Beilin LJ Irish AB . The effect of n-3 fatty acids and coenzyme Q10 supplementation on neutrophil leukotrienes, mediators of inflammation resolution and myeloperoxidase in chronic kidney disease. Prostaglandins Other Lipid Mediat. (2018) 136:1–8. doi: 10.1016/j.prostaglandins.2018.03.002, 29577973

[144] Wu W Chen F Ma H Lu J Zhang Y Zhou H . Dietary protein requirements of older adults with sarcopenia determined by the indicator amino acid oxidation technology. Front Nutr. (2025) 12:1486482. doi: 10.3389/fnut.2025.1486482, 40093878 PMC11906324

[145] Nishimura Y Højfeldt G Breen L Tetens I Holm L. Dietary protein requirements and recommendations for healthy older adults: a critical narrative review of the scientific evidence. Nutr Res Rev. (2023) 36:69–85. doi: 10.1017/s0954422421000329 34666855

[146] Koh A Molinaro A Ståhlman M Khan MT Schmidt C Mannerås-Holm L . Microbially produced imidazole propionate impairs insulin Signaling through mTORC1. Cell. (2018) 175:947–961.e17. doi: 10.1016/j.cell.2018.09.055, 30401435

[147] Sorgdrager FJH Naudé PJW Kema IP Nollen EA Deyn PP. Tryptophan metabolism in inflammaging: from biomarker to therapeutic target. Front Immunol. (2019) 10:2565. doi: 10.3389/fimmu.2019.02565, 31736978 PMC6833926

[148] Kaiser H Yu K Pandya C Mendhe B Isales CM McGee-Lawrence ME . Kynurenine, a tryptophan metabolite that increases with age, induces muscle atrophy and lipid peroxidation. Oxidative Med Cell Longev. (2019) 2019:1–9. doi: 10.1155/2019/9894238, 31737181 PMC6815546

[149] Scott SA Fu J Chang PV. Microbial tryptophan metabolites regulate gut barrier function via the aryl hydrocarbon receptor. Proc Natl Acad Sci USA. (2020) 117:19376–87. doi: 10.1073/pnas.2000047117, 32719140 PMC7431026

[150] Shi D Cui Y Liang H Wei Q Huang J Cao J. Gut microbiota-derived tryptophan metabolite indole-3-carboxaldehyde enhances intestinal barrier function via aryl hydrocarbon receptor/AMP-activated protein kinase signaling activation. Anim Biosci. (2026) 39:250225. doi: 10.5713/ab.25.0225, 40665732 PMC12754468

[151] Blachier F Beaumont M Kim E. Cysteine-derived hydrogen sulfide and gut health: a matter of endogenous or bacterial origin. Curr Opin Clin Nutr Metab Care. (2019) 22:68–75. doi: 10.1097/mco.0000000000000526, 30461448

[152] Zhong VW Van Horn L Greenland P Carnethon MR Ning H Wilkins JT . Associations of processed meat, unprocessed red meat, poultry, or fish intake with incident cardiovascular disease and all-cause mortality. JAMA Intern Med. (2020) 180:503–12. doi: 10.1001/jamainternmed.2019.6969, 32011623 PMC7042891

[153] Berry SE Valdes AM Drew DA Asnicar F Mazidi M Wolf J . Human postprandial responses to food and potential for precision nutrition. Nat Med. (2020) 26:964–73. doi: 10.1038/s41591-020-0934-0, 32528151 PMC8265154

[154] Rein M Ben-Yacov O Godneva A Shilo S Zmora N Kolobkov D . Effects of personalized diets by prediction of glycemic responses on glycemic control and metabolic health in newly diagnosed T2DM: a randomized dietary intervention pilot trial. BMC Med. (2022) 20:56. doi: 10.1186/s12916-022-02254-y, 35135549 PMC8826661

[155] Ben-Yacov O Godneva A Rein M Shilo S Kolobkov D Koren N . Personalized postprandial glucose response-targeting diet versus Mediterranean diet for Glycemic control in prediabetes. Diabetes Care. (2021) 44:1980–91. doi: 10.2337/dc21-0162, 34301736

[156] Bermingham KM Linenberg I Polidori L Asnicar F Arrè A Wolf J . Effects of a personalized nutrition program on cardiometabolic health: a randomized controlled trial. Nat Med. (2024) 30:1888–97. doi: 10.1038/s41591-024-02951-6, 38714898 PMC11271409

[157] Tily H Patridge E Cai Y Gopu V Gline S Genkin M . Gut microbiome activity contributes to prediction of individual variation in Glycemic response in adults. Diabetes Ther. (2022) 13:89–111. doi: 10.1007/s13300-021-01174-z, 34799839 PMC8776936

[158] Simon MC Sina C Ferrario PG Daniel H. Gut microbiome analysis for personalized nutrition: the state of science. Mol Nutr Food Res. (2023) 67:e2200476. doi: 10.1002/mnfr.202200476, 36424179

[159] Salminen S Collado MC Endo A Hill C Lebeer S Quigley EMM . The international scientific Association of Probiotics and Prebiotics (ISAPP) consensus statement on the definition and scope of postbiotics. Nat Rev Gastroenterol Hepatol. (2021) 18:649–67. doi: 10.1038/s41575-021-00440-6, 33948025 PMC8387231

[160] Kocsis T Molnár B Németh D Hegyi P Szakács Z Bálint A . Probiotics have beneficial metabolic effects in patients with type 2 diabetes mellitus: a meta-analysis of randomized clinical trials. Sci Rep. (2020) 10:11787. doi: 10.1038/s41598-020-68440-1, 32678128 PMC7366719

[161] Li G Feng H Mao XL Deng YJ Wang XB Zhang Q . The effects of probiotics supplementation on glycaemic control among adults with type 2 diabetes mellitus: a systematic review and meta-analysis of randomised clinical trials. J Transl Med. (2023) 21:442. doi: 10.1186/s12967-023-04306-0, 37415167 PMC10324246

[162] Li D Lu Y Yuan S Cai X He Y Chen J . Gut microbiota-derived metabolite trimethylamine-N-oxide and multiple health outcomes: an umbrella review and updated meta-analysis. Am J Clin Nutr. (2022) 116:230–43. doi: 10.1093/ajcn/nqac074, 35348578 PMC9257469

[163] Farhangi MA Vajdi M Asghari-Jafarabadi M. Gut microbiota-associated metabolite trimethylamine N-oxide and the risk of stroke: a systematic review and dose-response meta-analysis. Nutr J. (2020) 19:76. doi: 10.1186/s12937-020-00592-2, 32731904 PMC7393891

[164] Wang B Qiu J Lian J Yang X Zhou J. Gut metabolite trimethylamine-N-oxide in atherosclerosis: from mechanism to therapy. Front Cardiovasc Med. (2021) 8:723886. doi: 10.3389/fcvm.2021.723886, 34888358 PMC8650703

[165] Jing L Zhang H Xiang Q Shen L Guo X Zhai C . Targeting trimethylamine N-oxide: a new therapeutic strategy for alleviating atherosclerosis. Front Cardiovasc Med. (2022) 9:864600. doi: 10.3389/fcvm.2022.864600, 35770223 PMC9235870

[166] Argyridou S Davies MJ Biddle GJH Bernieh D Suzuki T Dawkins NP . Evaluation of an 8-week vegan diet on plasma trimethylamine-N-oxide and Postchallenge glucose in adults with Dysglycemia or obesity. J Nutr. (2021) 151:1844–53. doi: 10.1093/jn/nxab046, 33784394 PMC8245890

[167] Cohen PA. Probiotic safety-no guarantees. JAMA Intern Med. (2018) 178:1577–8. doi: 10.1001/jamainternmed.2018.5403, 30242393

